# Effects of vector control interventions on spatio-temporal changes of *falciparum* malaria risk in children aged 2–10 in sub-Saharan African regions during 2011–2020

**DOI:** 10.3389/fpubh.2025.1531771

**Published:** 2025-07-02

**Authors:** Changkuoth Jock Chol, Denekew Bitew Belay, Haile Mekonnen Fenta, Ding-Geng Chen

**Affiliations:** ^1^Department of Statistics, College of Science, Bahir Dar University, Bahir Dar, Ethiopia; ^2^Department of Statistics, College of Natural and Computational Science, Gambella University, Gambella, Ethiopia; ^3^Department of Statistics, University of Pretoria, Pretoria, South Africa; ^4^Center for Environmental and Respiratory Health Research, Population Health, University of Oulu, Oulu, Finland; ^5^Biocenter Oulu, University of Oulu, Oulu, Finland; ^6^College of Health Solutions, Arizona State University, Tempe, AZ, United States

**Keywords:** autocorrelation, Bayesian, Markov-chain Monte Carlo, prevalence, vector control interventions

## Abstract

**Background:**

Sub-Saharan Africa (SSA) has a disproportionately high malaria fatality rate globally, with young children accounting for the majority of fatalities. The objective of this study is to investigate the spatiotemporal dynamics of malaria infection risk and assess the effect of vector control interventions on malaria infection rates in SSA nations.

**Methods:**

We utilized data from the Malaria Atlas Project regarding the prevalence of *Plasmodium falciparum* malaria infections and vector control interventions across 634 administrative areas in 45 SSA countries over a decade. This study adopted spatiotemporal regression models using Markov-chain Monte Carlo methods with a Bayesian setup.

**Results:**

Between 2011 and 2020, the average annual prevalence rates of malaria infection among children aged 2 to 10 in SSA diminished from 21.32% in 2011 to 16.75% in 2016, with a slight resurgence observed in 2017. Each unit increase in the number of individuals utilizing insecticide-treated nets (ITN) annually correlates with a 34.07% reduction in the risk of malaria infection. A rise in malaria cases has prompted SSA to undertake serious control measures. The auto-regressive process reveals a highly significant temporal correlation, while the global spatial dependency parameter indicates a modest spatial correlation. The highest risk of malaria infection prevalence among children aged 2 to 10 was indicated in states in the West-central, Central, and certain Eastern regions.

**Conclusion:**

Given that the West-central, Central, and select Eastern states exhibit the highest rates of malaria infection, the global end malaria councils and the malaria control and elimination program should prioritize interventions in these regions, enhancing vector control measures and providing comprehensive training on their effective utilization to mitigate malaria risk in these areas.

## 1 Introduction

Malaria claims a considerable number of lives. The most common malaria type in Sub-Saharan Africa (SSA) is *Plasmodium falciparum* malaria, which can be fatal. Severe malaria can have a 20% fatality rate (1). Infected blood cells with malaria from *Plasmodium* parasites cling to the endothelial lining of blood vessels, resulting in tissue damage and obstruction of the vessel (2). This infection causes a coma if it spreads to the brain (3). Respiratory failure may develop if the lungs are compromised; indeed, respiratory distress manifests in 40% of children and 25% of adults having serious malaria induced by *Plasmodium falciparum* (4). If the recipient is pregnant, the placenta can induce maternal anemia, early labor, a higher risk of stillbirth, and a small birth weight (2). In SSA, gestational malaria collectively results in up to 200,000 infant deaths annually (5).

Africa accounts for a disproportionate share of global malaria fatalities, mostly due to the prevalence of *Plasmodium falciparum* malaria (6). Malaria mortality has been steadily decreasing outside of Africa since the 1980s, but it has been increasing within the continent, with 1.613 million fatalities in 2004 (7). Since that time, malaria fatalities have decreased, however they remain significantly higher than in other places (8). In 2019, malaria resulted in approximately 558,000 fatalities globally, with 534,000 occurring in Africa (9). The WHO reports that 96% of the 627,000 malaria fatalities globally in 2020 transpired in SSA, resulting in 602,000 deaths from the disease in that region (9). This rise is ascribed to the *coronavirus* pandemic, which jeopardised the availability of malaria control services such as indoor residual spraying (IRS) and insecticide-treated bed nets (10). The majority of malaria deaths occur in children under 5 years old (11), with children ages one to three being the most affected. According to Mbishi et al. (12), children under 5 years in SSA nations were still at risk for malaria.

Following World War II, the introduction of pesticides like *dieldrin* (DLD) and *dichloro-diphenyl-trichloroethane* (DDT) led to a sharp decline in malaria cases in various regions of Africa (13). By the 1950s, malaria had been eliminated in the United States (14). In this time, the cost of the antimalarial drugs *chloroquine* fell, resulting in widespread use throughout Africa during the 1960s and 1970s. Even so, *chloroquine*-resistant parasites began to emerge in the 1970s, and as of the 1980s, disease was once again spreading (13). Since the early 2000s, combination therapy based on *artemisinin*, including *artemisinin* and many other drugs, has been widely recognized as the most effective treatment for malaria (15).

Okumu (2020) states that the use of pesticide-covered mosquito nets can reduce mosquito exposure to malaria by 25–30% (16). In historical and program documentation, IRS has also been shown to help reduce malaria; however, randomized control studies have not consistently shown IRS to be an effective strategy (17). Recent developments in control efforts have led to numerous advances, including quick diagnostic tests and especially effective medications, including the artemisinin combination therapy. Increased use of insecticide-treated bed nets, various vector control techniques, and preventative intermittent chemotherapy treatments for individuals at risk have all contributed to a decrease in the incidence of malaria (18).

Trends combine mapping, spatiotemporal modeling, and storytelling to bring the global issue of malaria closer to different audiences. This tool provides malaria risk, burden, and intervention data through maps, graphs, and tables to easily visualize and explore trends in malaria and related topics at different geographic scales. Even though only a few studies (19–21) have been conducted on the effects of vector control interventions on changes in the incidence of malaria *parasitemia* in various countries, none of the studies used spatiotemporal data nature ranging more than 2 years, and there is a lack of broad knowledge regarding the disease at the SSA regional level. The purpose of this study is to evaluate the effects of vector control initiatives on malaria infection risk at 634 sub-national levels in 45 SSA nations among children aged 2 to 10, from 2011 to 2020, as well as to estimate spatiotemporal patterns of malaria infection risk changes. The study’s findings will shed light on the effectiveness of actions, and the National Malaria Control Program (NMCP) in SSA countries and the Ministry of Health (MoH) will utilize them to review programs and allocate resources most effectively to achieve their objectives.

## 2 Materials and methods

### 2.1 Settings

**Figure 1:**
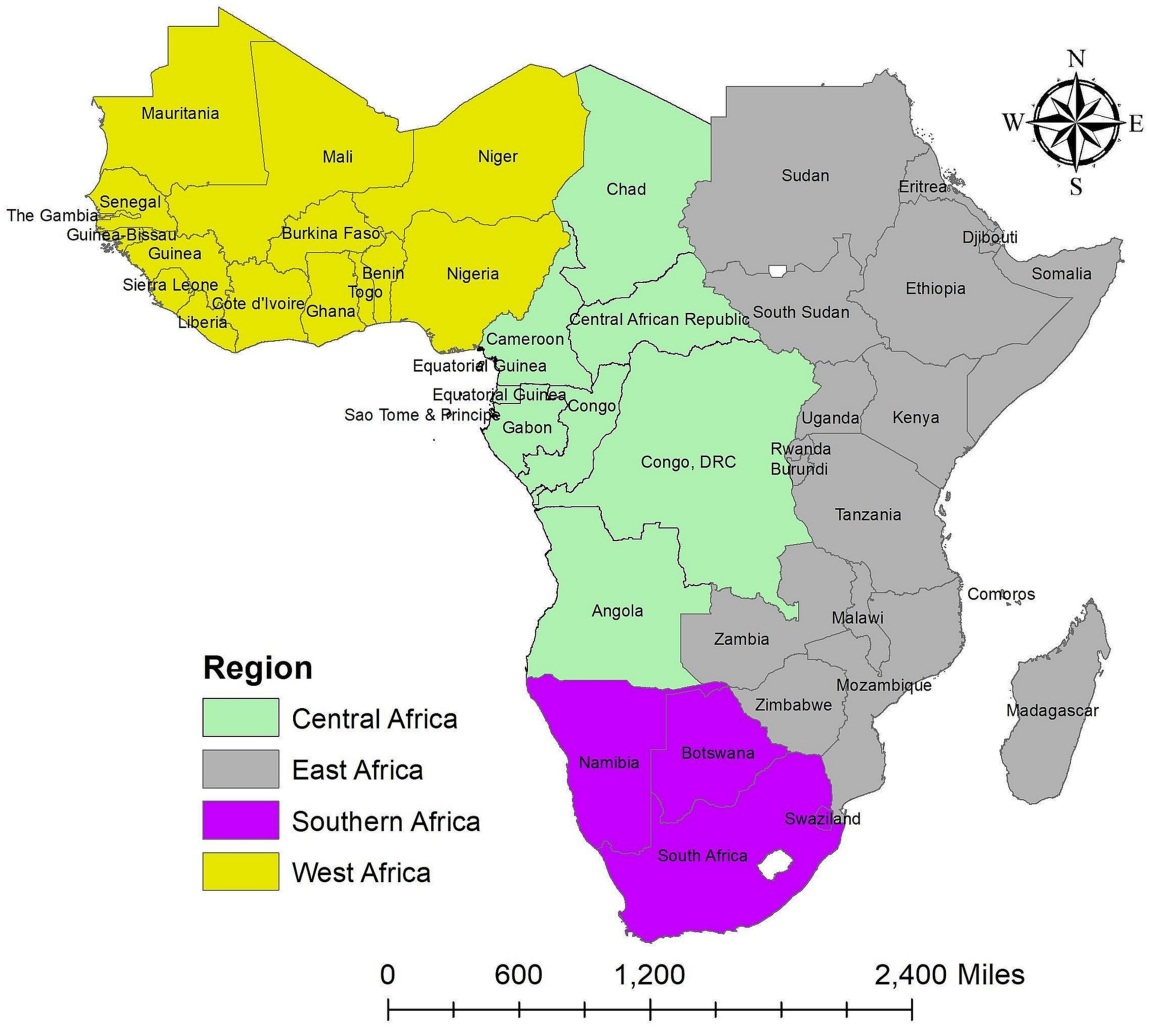
Map of the study area. Source of shapefile: Database of Global Administrative Areas v.4.1 (www.gadm.org), own map output from ArcGIS v.10.8 (https://desktop.arcgis.com).

This study was carried out in 634 administration level 1 (sub-national level) that received funding for vector control programs (Insecticide-Treatment Net (ITN), Indoor Residual Spraying (IRS), and Antimalarial Effective Treatment) over the study period (2011–2020) in 45 SSA countries settings to quantify the temporal and spatial distribution of changes in malaria infection risk and evaluate the influence of vector control initiatives on the risk of malaria infection at 634 sub-national levels in 45 SSA countries among children aged 2–10 between 2011 and 2020. SSA refers to the African continent territories south of the Sahara Desert. The regions of Central Africa, East Africa, Southern Africa, and West Africa make up the SSA (Figure 1). Although the SSA countries go by different titles for administration level 1, for the sake of this study, they were all referred to as states. The Supplementary Figure S1 and Supplementary Table S1 contained the study area’s map and names for each state.

### 2.2 Data sources

The Malaria Atlas Project Data Platform provides several tools for studying, examining, and interacting with malaria data (22). The portal also includes malaria data to varying degrees of detail to meet different demands. We used a recently published database of *Plasmodium falciparum* clinical infection prevalence and vector control intervention data in the SSA obtained from the Malaria Atlas Project Data Platform website. ^1^ For our models, we used the estimates of malaria infection prevalence for *Plasmodium falciparum* parasite rate to the age group 2–10 years old $\left(\mathrm{PfP}R_{2-10}\right)$ , as *Plasmodium falciparum* malaria has the highest mortality in children in the SSA. We aggregated data by averaging $\left(\mathrm{PfP}R_{2-10}\right)$ at the first administrative level within a country in the SSA (i.e., the state in this study) between 2011 and 2020, using shape files provided by the Database of Global Administrative Areas dataset version 4.1. ^2^ We used the proportion of malaria infection for $\left(\mathrm{PfP}R_{2-10}\right)$ (per 100 children) as a dependent variable and the coverage of malaria vector control interventions as covariates (Table 1).

**Table 1:** Intervention covariates.

Name	Metric	Definition
ITN use (in 100 people)	Use	The proportion of the population that sleeps under an Insecticide-Treated Net during a defined year
ITN access (in 100 people)	Access	The proportion of the population with access to an Insecticide-Treated Net in their household during a defined year
ITN use rate (in 100 people)	Use Rate	The proportion of people sleeping under an Insecticide-Treated Net, among those with access to an Insecticide-Treated Net in their household during a defined year
IRS coverage (in 100 households)	Coverage	The proportion of households that received indoor residual spraying in a given year
Treatment (in 100 malaria cases)	Antimalarial Effective Treatment	The proportion of malaria cases that receive effective treatment with antimalarial medicine

### 2.3 Statistical models

This study is based on the *Plasmodium falciparum* malaria prevalence for children aged 2 to 10 years old in the SSA from 2011 to 2020. First, we used Anselin Local Moran’s I statistic (local indicators of spatial association [LISA]) to quantify it on a local scale. The LISA statistics were used to detect malaria clustering and locate hotspots. The features of ArcMap software version 10.8 were used to conduct these investigations. Multiple options exist to model spatiotemporal data related to the different coverage of malaria vector control intervention data sets because SSA countries have 634 administrative states and areal units. Here, we present the relevant covariates and analyze the autoregressive (AR) model, developed by Rushworth et al. (23) which describes the spatiotemporal pattern in the mean response by using a single set of geographically and temporally autocorrelated random effects. These effects follow a multivariate autoregressive process of order 1 or 2. Allowable data models are binomial, Gaussian, and Poisson (24).

There are various determinants of the effect of interventions on *Plasmodium falciparum* malaria prevalence risk. These include the proportion of people who sleep under ITN, the proportion of people who have access to ITN in their homes, the proportion of people who sleep under ITN among those who have access to ITN in their homes, the proportion of households that have IRS coverage, and the proportion of malaria cases that are successfully treated with antimalarial medication. We define treatment as the proportion of malaria cases that were successfully treated with antimalarial medications. It is possible that states with high or low rates of malaria infection also have the highest or lowest rates of effective antimalarial medication. The response variable of this study is the proportion of malaria infection for $\mathrm{PfP}R_{2-10}$ (per 100 children). The Malaria Atlas Project Data Platform provides only the proportion of malaria infection for $\mathrm{PfP}R_{2-10}$ , we adopt the Gaussian distribution in our modeling. However, the observed response, being a proportion, is better modeled using a logit transformation in the first place. The logit transformed proportions are assumed to follow the normal error distribution in the Generalized Linear Models (GLM). Since the response is in the logit scale transform, we also logit transform the covariates.

#### 2.3.1 Spatio-temporal autoregressive model

Let $Y_{\mathrm{kt}}$ for k=1,..,634 and t=1,…,10 , denote the logit transformed estimated proportion of malaria infection for $\mathrm{PfP}R_{2-10}$ . In this study, n=634 is the number of administration areas (states) in the SSA countries and T=10 is the number of years we have data. The top-level model is specified as:


(1) $$Y_{\mathrm{kt}}=\log(\frac{{\hat{p}}_{\mathrm{kt}}}{1-{\hat{p}}_{\mathrm{kt}}})=x_{\mathrm{kt}}^{\prime }\beta +\psi_{\mathrm{kt}}+\in_{\mathrm{kt}},k=1,\ldots ,634,t=1,\ldots ,10,$$

where ${\hat{p}}_{\mathrm{kt}}$ is the estimated proportion of malaria infection for $\mathrm{PfP}R_{2-10}$ , $\in_{\mathrm{kt}}\sim N(0,v^{2})$ independently, $X_{\mathrm{kt}}=(x_{\mathrm{kt}1},\ldots ,x_{\mathrm{ktp}})$ is a vector of p control intervention covariates, $\beta =(\beta_{1},\ldots ,\beta_{p})$ is the vector of covariates regression parameter and $\psi_{\mathrm{kt}}$ are spatiotemporal random effects models (25, 26). In Equation (1), the basic linear model is obtained as a special case when $\psi_{\mathrm{kt}}=0$ for all values of k and t . In cases when both the dependent variable and the covariates are log-transformed variables in this study, the interpretation is given as the predicted percentage change in the dependent variable when the covariate increases by some percentage. To get the proportional change in Y associated with a p percent increase in X , we calculate α=log([100+p]/100) and take $e^{\alpha \hat{\beta }}$ linear-log. So that the percent change Y associated with percent increase in X is $100\times (e^{\alpha \hat{\beta }}-1)$ .

There are two versions of this model, which are based on either a first (AR(1)) or a second (AR(2)) order temporal autoregressive process. Rushworth et al. (23) presented the first-order model, which uses a multivariate first-order autoregressive process with a spatially autocorrelated precision matrix to characterize the spatiotemporal structure. This is expanded in the second model, which uses a spatially autocorrelated precision matrix and a multivariate second-order autoregressive process. If one wants to assess how the spatial structure in the data has changed over time, these models are suitable. Below are the model specifications for each scenario (Equation 2).

The AR(1) model specifies:


(2) $$\begin{array}{c} \psi_{\mathrm{kt}}=\phi_{\mathrm{kt}}\\ \phi_{t}|\phi_{t-1}\sim N(\rho_{T}\phi_{t-1},\tau^{2}Q{\left(W,\rho_{S}\right)}^{-1}),t=2,..,T\\ \begin{array}{c} \phi_{1}\sim N(0,\tau^{2}Q{\left(W,\rho_{S}\right)}^{-1})\\ \tau^{2}\sim \mathrm{IG}(a,b)\\ \rho_{S},\rho_{T}\sim \text{Unif}(0,1) \end{array} \end{array}$$

The AR(2) model specifies:


(3) $$\begin{array}{c} \psi_{\mathrm{kt}}=\phi_{\mathrm{kt}}\\ \phi_{t}|\phi_{t-1},\phi_{t-2}\sim N(\rho_{T}\phi_{t-1}+\rho_{T}\phi_{t-2},\tau^{2}Q{\left(W,\rho_{S}\right)}^{-1}),t=3,..,T\\ \begin{array}{c} \phi_{1},\phi_{2}\sim N(0,\tau^{2}Q{\left(W,\rho_{S}\right)}^{-1})\\ \tau^{2}\sim \mathrm{IG}(a,b)\\ \begin{array}{c} \rho_{S}\sim \text{Unif}(0,1)\\ f(\rho_{T_{1}},\rho_{T_{2}})\propto 1 \end{array} \end{array} \end{array}$$

Here $\phi_{t}=(\phi_{1t},\ldots ,\phi_{\mathrm{Kt}})$ is the vector of random effects for time t (Equation 3), which evolve via a multivariate first or second-order autoregressive process with temporal autoregressive parameter(s) $\rho_{T}$ (AR(1) model) or $\left(\rho_{T_{1}},\rho_{T_{2}}\right)$ (AR(2) model). The mean induces temporal autocorrelation, whereas the variance induces spatial autocorrelation $\tau^{2}Q{\left(W,\rho_{S}\right)}^{-1}$ . The corresponding precision matrix $Q(W,\rho_{S})$ was proposed by (27) and corresponds to the CAR models. This matrix’s algebraic form is provided by


(4) $$Q(W,\rho_{S})=\rho_{S}[\mathrm{diag}(W1)-W]+(1-\rho_{S})I,$$

where 1 is the K×1 vector of one while I is the K×K identity matrix (Equation 4). As with the other models, the random effects are zero-mean centered, and the default hyperparameter values are (a=1,b=0.01) , with flat and conjugate inverse-gamma priors provided for $\left(\rho_{S},\rho_{T},\rho_{T_{1}},\rho_{T_{2}}\right)$ and $\tau^{2}$ , respectively. The dependence parameters $\left(\rho_{S},\rho_{T}\right)$ can be fixed at values in the unit interval [0, 1] rather than being estimated in the model, while $\left(\rho_{T_{1}},\rho_{T_{2}}\right)$ can also be fixed.

#### 2.3.2 Model fitting, choice, and validation statistic

The models in this study are fitted in a Bayesian setting using Markov-chain Monte-Carlo simulation. Gibbs sampling is used for all parameters, including the variance and regression parameters (β) , whose entire conditional distributions have a closed-form distribution. Metropolis or Metropolis-Hastings Steps are used to update the remaining parameters.

We first compare four different models including the AR(2) model. In this study, the independent error regression model is the first model, followed by the ANOVA model (28), the AR(1) model, and the AR(2) model. To compare the studied models, we use the Deviance Information Criterion (DIC) (29) and the Watanabe Akaike information criterion (WAIC) (30), two widely used criteria to compare models in a fully Bayesian setting. The model with the smallest value of DIC and WAIC is the one with a better balance between the model adjustment and complexity. However, it is reassuring to see that the penalty parameters are estimated to be positive.

In this study, we selected only the four most widely used model validation criteria, especially for prediction using spatiotemporal modeling. These are Root Mean Square Error (RMSE), Mean Absolute Error (MAE), Continuous Ranked Probability Score (CRPS), and Coverage (CVG) to see the best goodness-of-fit, the smallest value is the best goodness-of-fit. However, CVG is not a discrepancy measure like the other three criteria. The theoretical value of 100(1−α) will be the optimal value. The model that produces a CVG value closest to 100(1−α) is to be chosen as the best model. The model validation is performed automatically by specifying the optional vector-valued (valid-rows) argument containing the row number of the data frame (26). In this study, 10 % of the data set was used for model validation using the *CARBayesST* package in R version 4.4.1 for the additional argument-valid rows supplied.

We do a permutation test for every year of data independently and compute Moran’s I statistic (31) to measure the existence of spatial autocorrelation in the residuals from this model. The alternative hypothesis of the permutation test indicates significant spatial autocorrelation, while the null hypothesis is that there is no spatial autocorrelation.

## 3 Results

### 3.1 Descriptive statistics results

**Figure 2:**
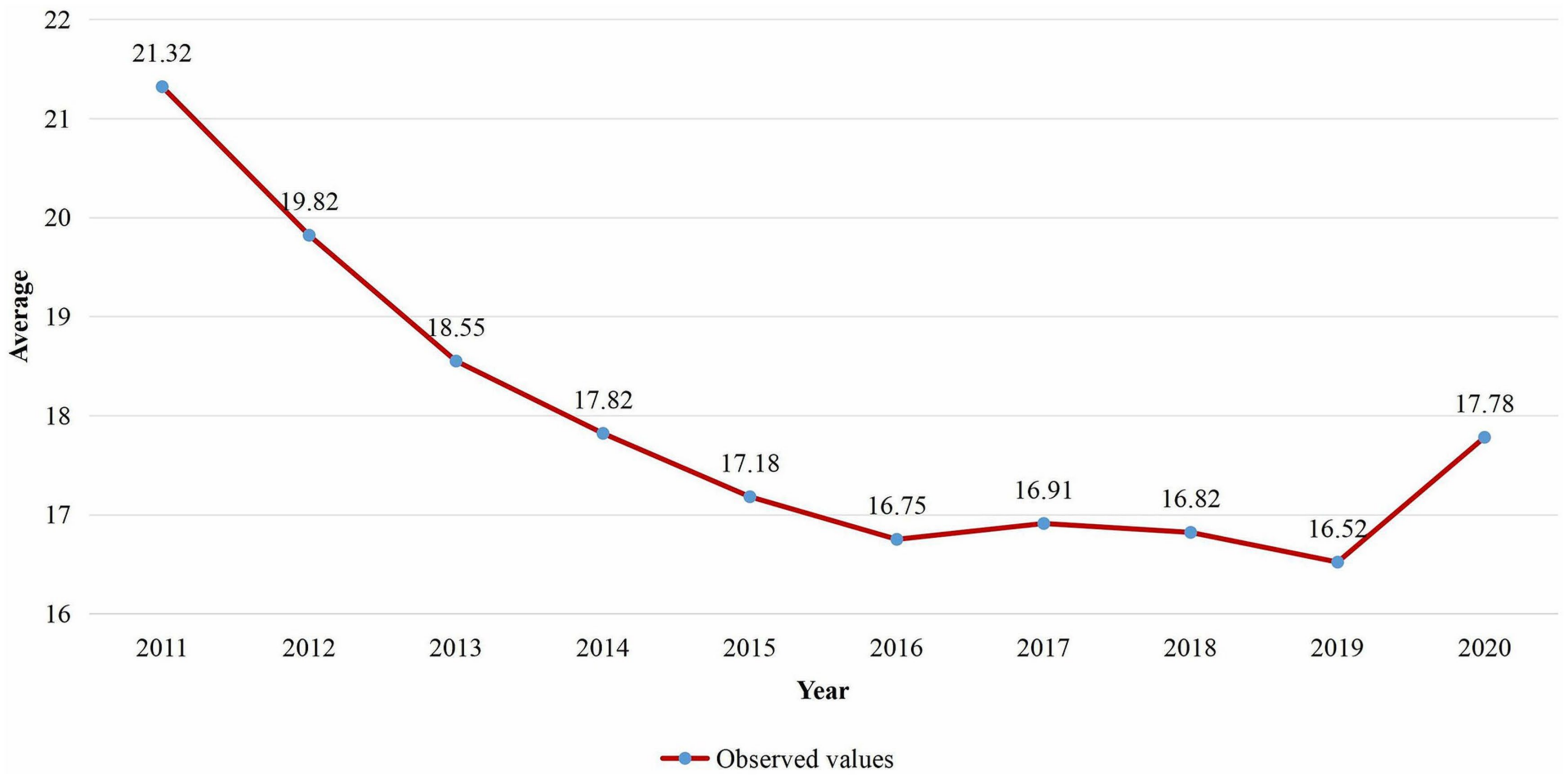
Temporal trend of the average proportion of malaria infection for $\mathrm{PfP}R_{2-10}$ in SSA from 2011 to 2020.

The yearly average proportion of malaria infection for $\mathrm{PfP}R_{2-10}$ in the SSA worldwide from 2011 to 2020 was presented in Figure 2. According to the findings, the proportion of malaria infection for $\mathrm{PfP}R_{2-10}$ dropped from an average of 21.32 in 2011 to 16.75 in 2016, with a minor increase observed between 2016 and 2017. However, it dropped from 16.91 in 2017 to 16.52 in 2019 and then rose to an average of 17.78 in 2020.

**Figure 3:**
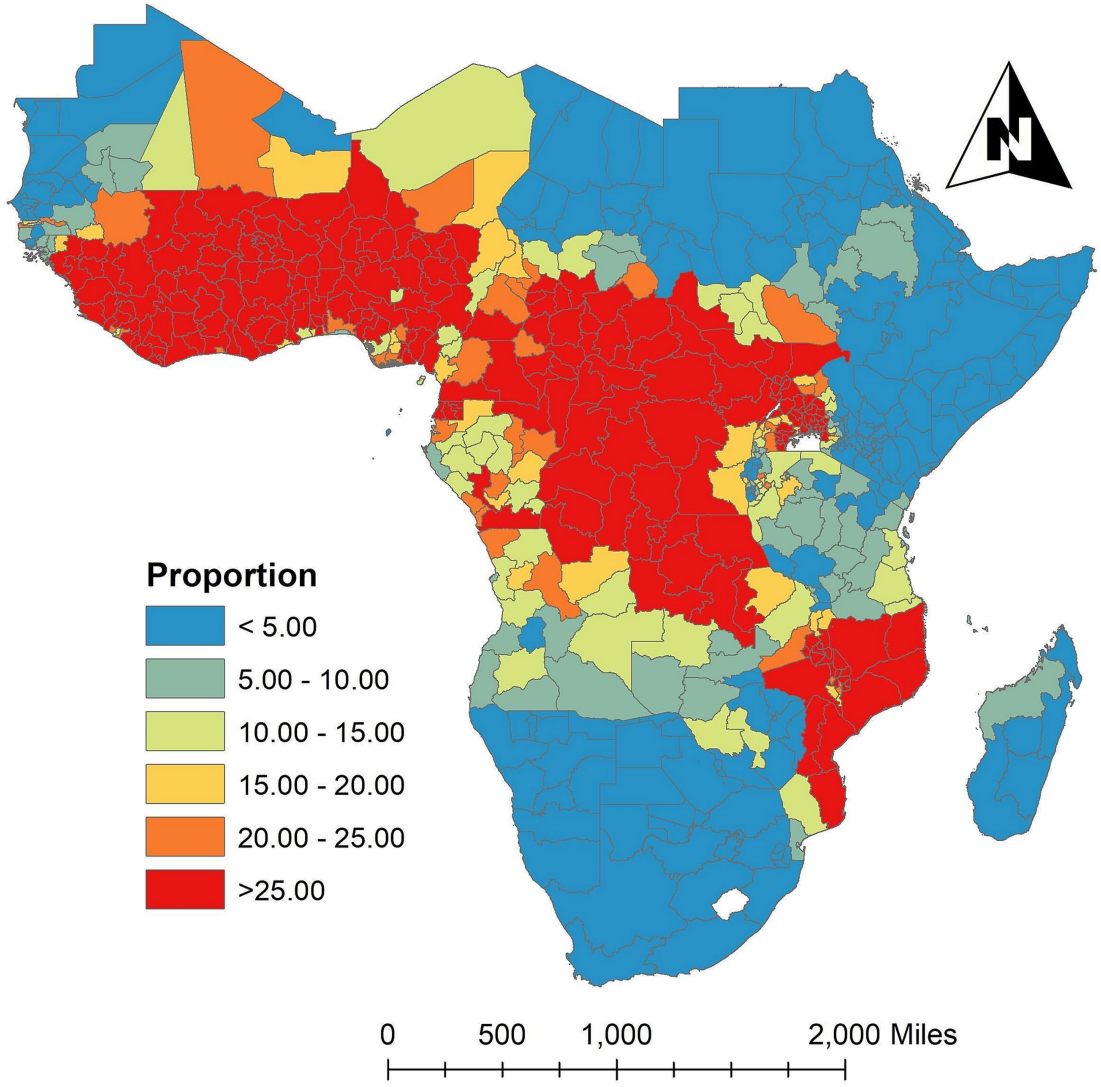
Spatially aggregated the proportion of malaria infection for $\mathrm{PfP}R_{2-10}$ in the SSA over the study period. Source of shapefile: Database of Global Administrative Areas v.4.1 (www.gadm.org), own map output from ArcGIS v.10.8 (https://desktop.arcgis.com).

The risk of *Plasmodium falciparum* parasite malaria infection among children aged 2 to 10 in each state is depicted in Figure 3, which is the geographic distribution aggregated of the averaged proportion of malaria infection for $\mathrm{PfP}R_{2-10}$ in the SSA across the study period. According to the results, the states around the West-central, Central, and certain Eastern states have the highest proportion of malaria infection for $\mathrm{PfP}R_{2-10}$ , while the states surrounding the Southern, Horn of Africa, and Northwest regions in SSA have the lowest frequency. Between 2011 (baseline year) and 2020 (endline year), the Supplementary Figure S2 showed changes in 634 administrative regions, including the percentage change in malaria prevalence and the number of locations where the proportion increased, decreased, or remained stable.

**Figure 4:**
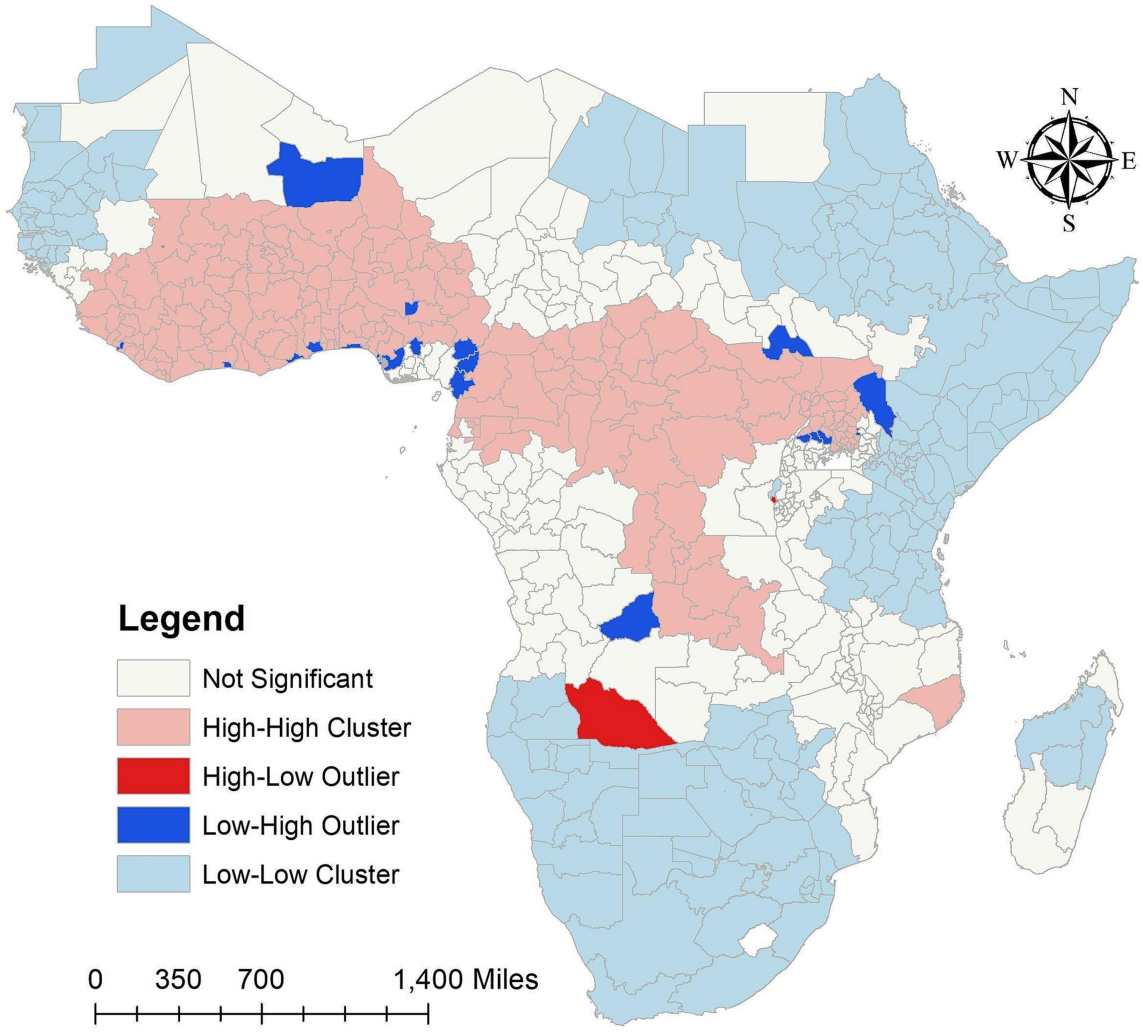
Cluster and outlier analysis malaria infection for $\mathrm{PfP}R_{2-10}$ in the SSA over the study period (2011–2020). Source of shapefile: Database of Global Administrative Areas v.4.1 (www.gadm.org), own map output from ArcGIS v.10.8 (https://desktop.arcgis.com).

In order to determine which states have a high number of malaria infections for $\mathrm{PfP}R_{2-10}$ in the SSA over the study period while their neighbors have low numbers, or which states have low numbers of malaria infections while their neighbors have high numbers of malaria infections, Figure 4 in this study shows the results that identify states that either have higher or lower malaria infections than the neighboring areas. It also identifies outlier states that are significantly different from their neighbors. According to the results, the SSA high cluster areas over the study period were the West-central, Central, and some parts of the Southeast, whereas the Northwest, Northeast, and certain sections of the eastern and southern states were low cluster areas. However, the states around North Madagascar were low cluster areas, and the South of Madagascar remained insignificant.

In our study findings, over the study period the Low-High outliers states are Bamako and Gao in Mali, Montserrado in Liberia, Abidjian in Côte d’Ivoire, Greater Accra in Ghana, Maritime in Togo, Lagos, Delta, Enugu and Federal Capital Territory (Abuja) in Nigeria, Nord-Ouest and Ouest in Cameroon, Littorial in Equatorial Guinea, Lunda Sul in Angola, Lakes in South Sudan, Turkana in Kenya, and Sironko, Kibale, Kiboga and Luwera in Uganda. However, high-low outliers states of malaria infections for $\mathrm{PfP}R_{2-10}$ in the SSA over the study period are Cuando Cubango in Angola, and Cibitoke in Burundi (Figure 4). The high cluster, low cluster, low-high and high-low outliers areas results of malaria infections for $\mathrm{PfP}R_{2-10}$ in the SSA from 2011 to 2020 were provided in the Supplementary Figure S3.

**Figure 5:**
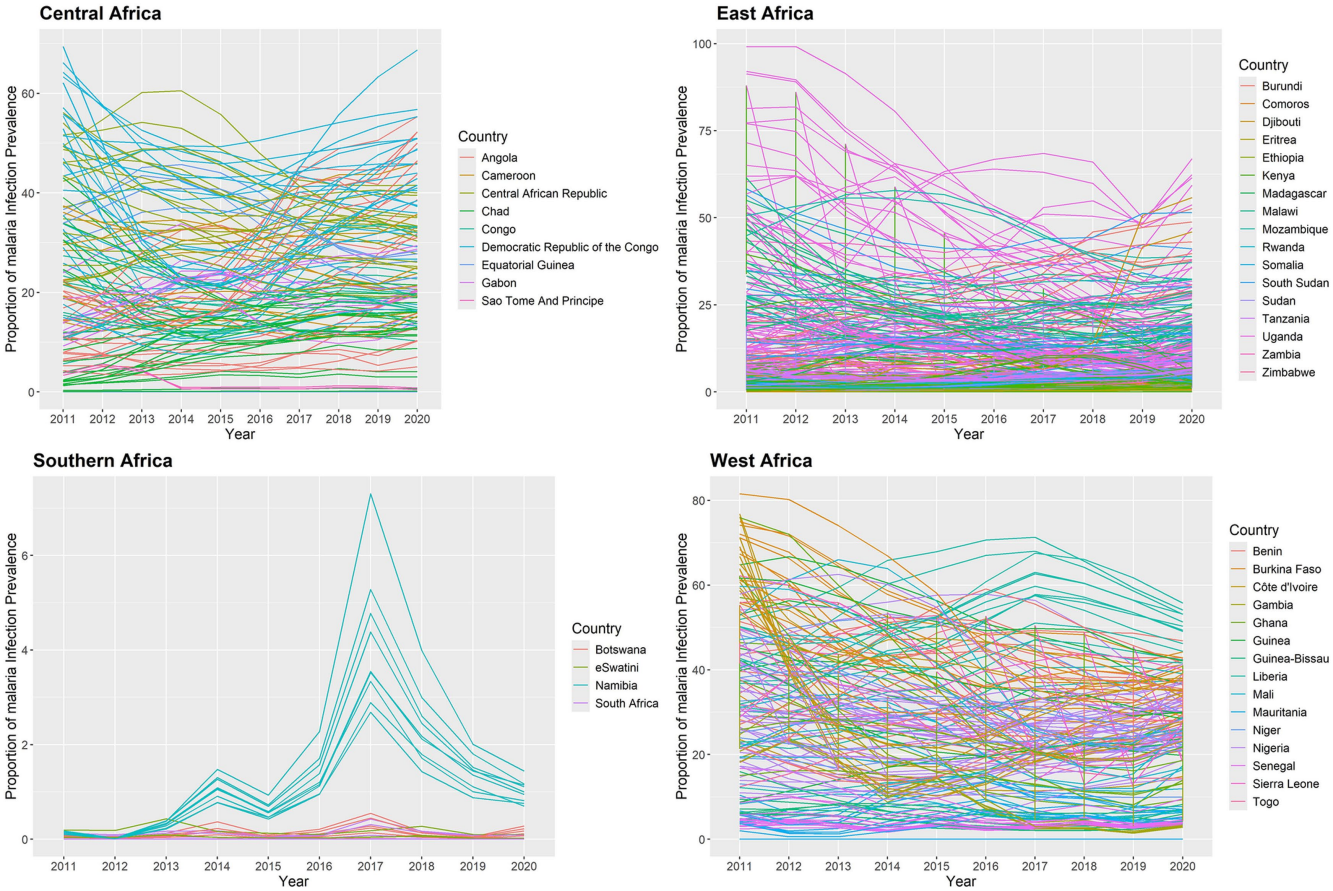
Temporal trend of the proportion of malaria infection for $\mathrm{PfP}R_{2-10}$ in the SSA at each state from 2011 to 2020.

The estimated yearly proportion of malaria infection for $\mathrm{PfP}R_{2-10}$ of 634 states in the SSA regions between 2011 and 2020 is shown in Figure 5. The findings indicate that between 2011 and 2020, the proportion of malaria infection varied over time in each state in SSA. The results showed that between 2011 and 2020 the proportion of malaria infection was high in states around the Western and Central regions and low in states surrounding the Southern and some Eastern areas. Nonetheless, a few states in the Eastern area exhibit a decline between 2011 and 2019. During the study period, certain states in the Central African Republic showed declines, while other states in the region around Gabon and the Democratic Republic of the Congo showed significant rises. Nonetheless, during the study period, malaria infections decreased in numerous states in Uganda. Furthermore, during the study period, malaria infection rates were rising in the states surrounding Namibia and Botswana in 2014 and 2017.

According to the results, there was strong evidence of unexplained spatial autocorrelation in the residuals from 2011 to 2020 after adjusting for the effects of the intervention covariates. The annual proportion of malaria infections had positive autocorrelation from 2011 to 2020 in the SSA global, ranging from 0.42851 to 0.50947, and all *p*-values were less than 0.05 (Table 2).

**Table 2:** Global Moran’s I autocorrelation values of annual malaria infection prevalence rates in the SSA.

Year	Moran’s I	*p*-value
2011	0.43991	0.001
2012	0.42851	0.001
2013	0.45083	0.001
2014	0.44329	0.001
2015	0.45928	0.001
2016	0.47630	0.001
2017	0.48364	0.001
2018	0.47112	0.001
2019	0.50297	0.001
2020	0.50947	0.001

### 3.2 Spatiotemporal regression model results

Four different spatiotemporal regression models were fitted and compared. The linear trend model comes first, followed by the ANOVA model, the AR(1) model, and the AR(2) model. The DIC and WAIC criterion values for these models are shown in Table 3. The results show that the AR(1) model was selected based on DIC and WAIC. The DIC and WAIC criterion values of the AR(1) model are significantly lower than those of the other models. The AR(1) model’s DIC and WAIC are negative because the independent error variance $\left(v^{2}\right)$ is thought to be quite modest (Table 4).

**Table 3:** Model choice criteria values for spatiotemporal regression models.

Model	DIC	P.dic	WAIC	P.waic
Linear	−725.115	1222.736	−588.673	1132.426
ANOVA	4414.804	633.464	4432.229	592.610
AR(1)	−5506.542	4997.465	−6937.510	2603.101
AR(2)	−1584.215	4833.897	−2764.493	2669.930

**Table 4:** Parameter estimates from the spatiotemporal AR(1) model.

Coefficient	Mean	2.5%	97.5%
Intercept	−1.510	−1.774	−1.247
ITN use	−0.601	−0.704	−0.500
ITN access	0.428	0.323	0.535
ITN use rate	0.633	0.587	0.680
IRS coverage	0.019	0.007	0.030
Treatment	0.506	0.446	0.564
$v^{2}$	0.010	0.008	0.011
$\tau^{2}$	0.598	0.509	0.700
$\rho_{S}$	0.134	0.106	0.165
$\rho_{T}$	0.953	0.942	0.964

The validation results shown in Table 5 are based on 6,340 (=634 × 10) observations as we chose all 10 time points and 634 states for validation. Compared to the other models, the AR(1) model performs marginally better. The coverage value appears to be 97.634 for the 6,340 prediction intervals. The Supplementary material contains a plot of the predictions against the observed values (Supplementary Figure S4). The sites for which the y=x line displayed in blue, in the image does not appear in the 95% prediction intervals are indicated by the red-colored open circles. As a result, the analysis given above shows that AR(1) is the best model. Three concurrent Markov chains are used to run the AR(1) model; each runs 69,740 MCMC samples, with the first 6,340 samples eliminated as the burn-in period. 6,340 samples are available for inference after the data are thinned by 10 to lower the autocorrelation in the Markov chains. To make sure the Markov chains seem to have converged, we included the posterior distributions of all the parameters in the Supplementary Figures S5–S8, together with the trace plots and density estimates. The posterior distributions for parameters are centered near their real values (Table 4), and there is no evidence that the figures do not converge (Supplementary Figures S5–S8).

**Table 5:** Model validation criteria statistics.

Model	RMSE	MAE	CRPS	CVG
Linear	0.217	0.156	0.129	94.479
ANOVA	0.328	0.241	0.195	94.953
AR(1)	**0.185**	**0.106**	**0.176**	**97.634**
AR(2)	0.294	0.164	0.228	96.214

The bold value indicates the selected the model.

The selected AR(1) model’s parameter estimate is shown in Table 4. The model shows that each of the five intervention covariates is significant. ITN use is negatively significant, as predicted, suggesting that increased ITN use lowers the proportion of malaria infection in children aged 2 to 10 in SSA. If there are higher rates of malaria infection among children aged 2 to 10 in SSA, then ITN access, ITN use rate, IRS coverage, and effective treatment will all increase. This is because the coefficients of ITN access, ITN use rate, IRS coverage, and antimalarial effective treatment are all positively significant, even after controlling for spatiotemporal correlations in the data. The significance of fitting the geographic model is reaffirmed by the estimation that the spatial variance ( $\tau^{2}$ ) is greater than the independent error variance ( $v^{2}$ ). The global spatial dependency parameter ( $\rho_{S}$ ) indicates a moderate spatial correlation. In SSA, the auto-regressive process exhibits a significant temporal correlation ( $\rho_{T}$ ). The findings revealed that the spatial correlation $\rho_{S}$ is smaller than the temporal autocorrelation $\rho_{T}$ .

According to this study, the SSA risk of malaria infection among children ages 2 to 10 will drop by 0.60% for every 1% increase in the population sleeping under ITN each year. If the average yearly rate of malaria infection in SSA climbs by 0.43%, the proportion of population with access to ITN in their household during a defined year will be increase by one-percent. In addition, if the annual percentage of malaria infections in the SSA rises by 0.63%, the proportion of persons sleeping under ITN in households with access to ITN will rise by 1% during a given year. Furthermore, if the percentage of malaria infections in the SSA rises by 0.02% per year, the number of families covered by IRS rises by 1% within a given year. Moreover, if the annual average malaria infection rate in the SSA rises by 0.51%, the proportion of malaria cases receiving effective antimalarial therapy will rise by 1 % (Table 4).

**Figure 6:**
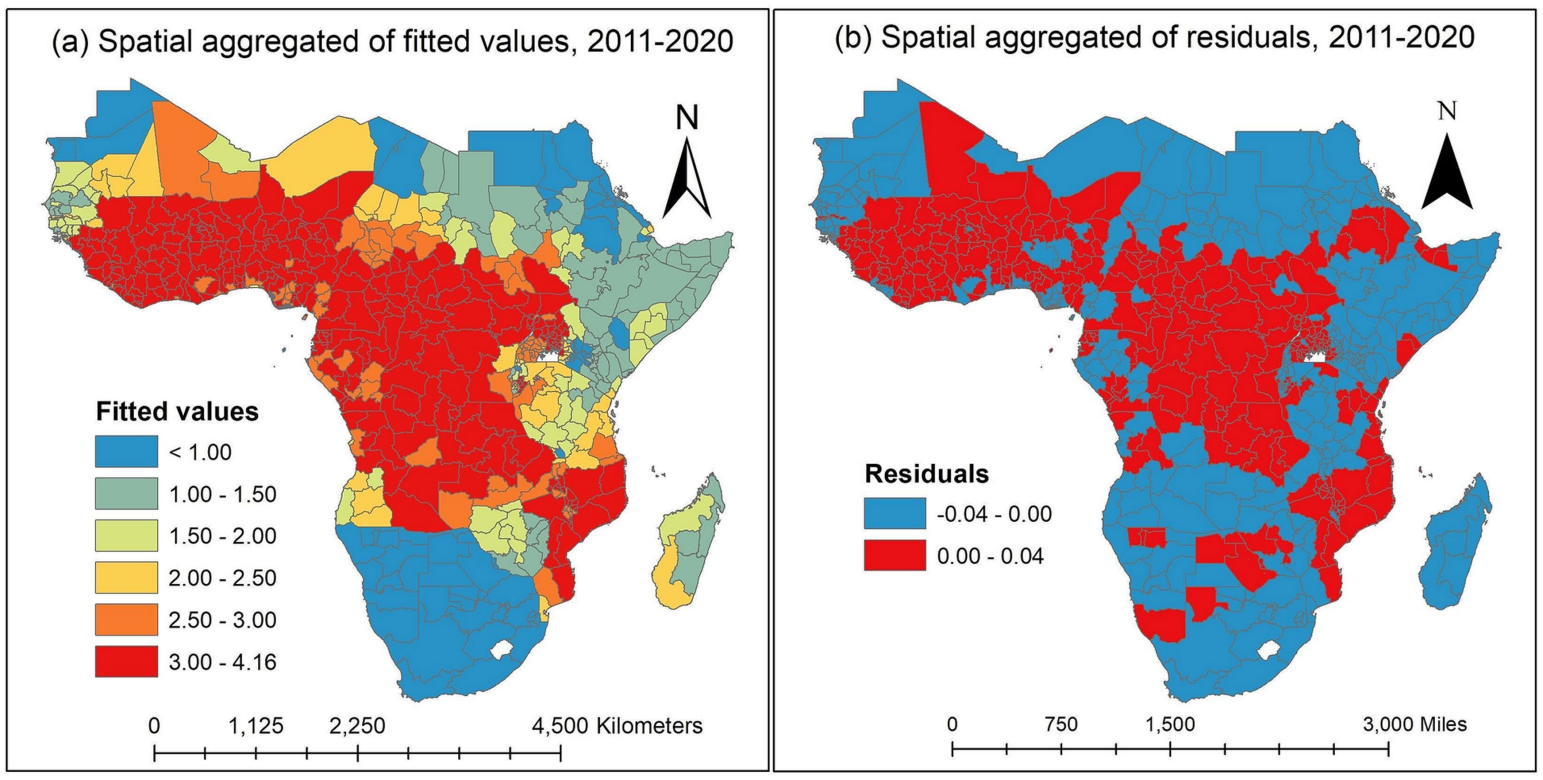
Spatially aggregated for posterior means of fitted values and residuals of the malaria infection prevalence rates over the study period in the SSA. **(a)** Spatially aggregated of fitted values. **(b)** Spatially aggregated of residuals. Source of shapefile: Database of Global Administrative Areas v.4.1 (www.gadm.org), own map output from ArcGIS v.10.8 (https://desktop.arcgis.com).

The spatial aggregation of fitted values of the proportion of malaria infection response variable throughout the study is plotted in Figure 6a. The fitted map and the observed map in Figure 3 are in excellent agreement. The discretized color categories do not match in a small number of states, perhaps because of the discretization process itself. The results showed that the states in the West-central, Central, and certain Eastern regions had the highest risk of malaria infection among children aged 2 to 10. However, the Northwest, Southern, and Horn of Africa states have the lowest risk of malaria infection. The states with the highest risk of malaria infection among children aged 2 to 10 years were those surrounding Equatorial Guinea, Cameroon, Angola, Congo, Gabon, Central African Republic, and the Democratic Republic of Congo in the Central region; South Sudan, Uganda, East Burundi, Malawi, Zambia, and Mozambique in the Eastern region; and Guinea, Sierra Leone, Liberia, Mali, Côte d’Ivoire, Burkina Faso, Ghana, Togo, Benin, Niger, and Nigeria in the Western region with credible intervals of posterior means ranging between 2.00 and 4.16 (Figure 6a).

The spatially aggregated residuals of the proportion of malaria infection in the SSA during the study period are displayed in Figure 6b. We examined the residuals to seek any discernible spatial pattern provided to the spatial map. For every observed data point, we acquire the so-called response residuals (observed-fitted) to create the spatial residual map. There are no overwhelming spatial patterns in the residual map indicating a need for additional investigation.

**Figure 7:**
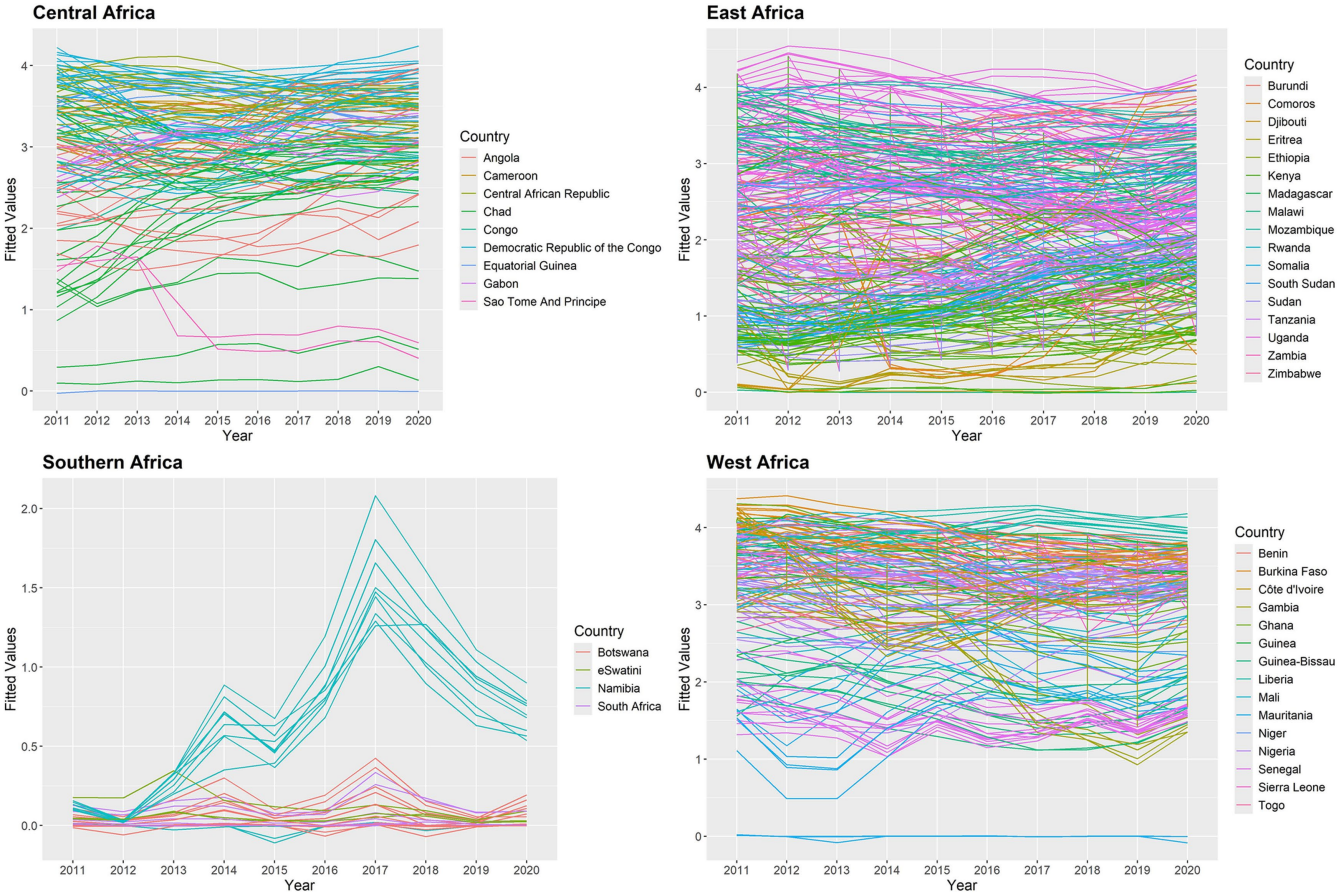
Posterior mean of fitted values for temporal trend for malaria infection prevalence rates in the SSA at the state level.

In this study, we used the temporal trend for the posterior mean of fitted values to estimate the yearly averages of 634 states in the SSA between 2011 and 2020. The temporal trend for the fitted values of the annual proportion of malaria infections is shown in Figure 7. The results indicate that during the study period, each state had a different risk value for the yearly proportion of malaria infections in children aged 2 to 10 years due to the *Plasmodium falciparum* parasite. Between 2011 and 2020, the states around the Central, Western, and certain Eastern states had the highest risk of contracting malaria for $\mathrm{PfP}R_{2-10}$ , while the states surrounding the Southern region had the lowest risk compared to other regions. Malaria infections increased in a few Southern states between 2012 and 2017 and fell between 2017 and 2020.

**Figure 8:**
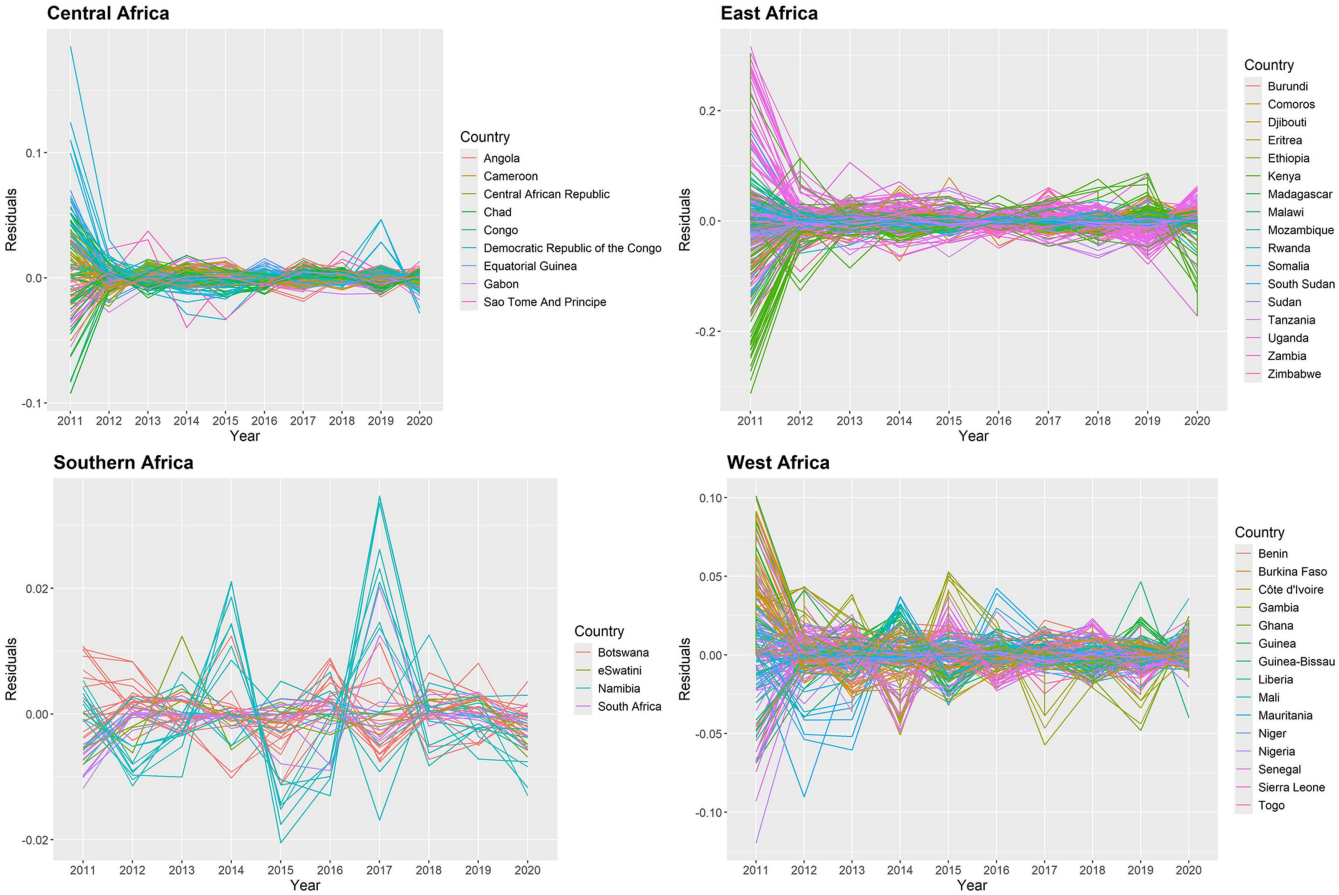
Posterior mean of residuals for temporal trend for malaria infection prevalence rates in the SSA at the state level.

We check the AR(1) model’s residuals to see whether there are any overwhelming temporal dependencies of 634 states from 2011 to 2020 in the SSA. The residuals are plotted in Figure 8, and it is evident that the temporal patterns are not displayed in this plot. In 2011, there were very few significant data points in the East Africa region; yet the related residuals’ absolute values were significantly smaller. As a result, it is believed that the AR(1) model fits the data well.

**Figure 9:**
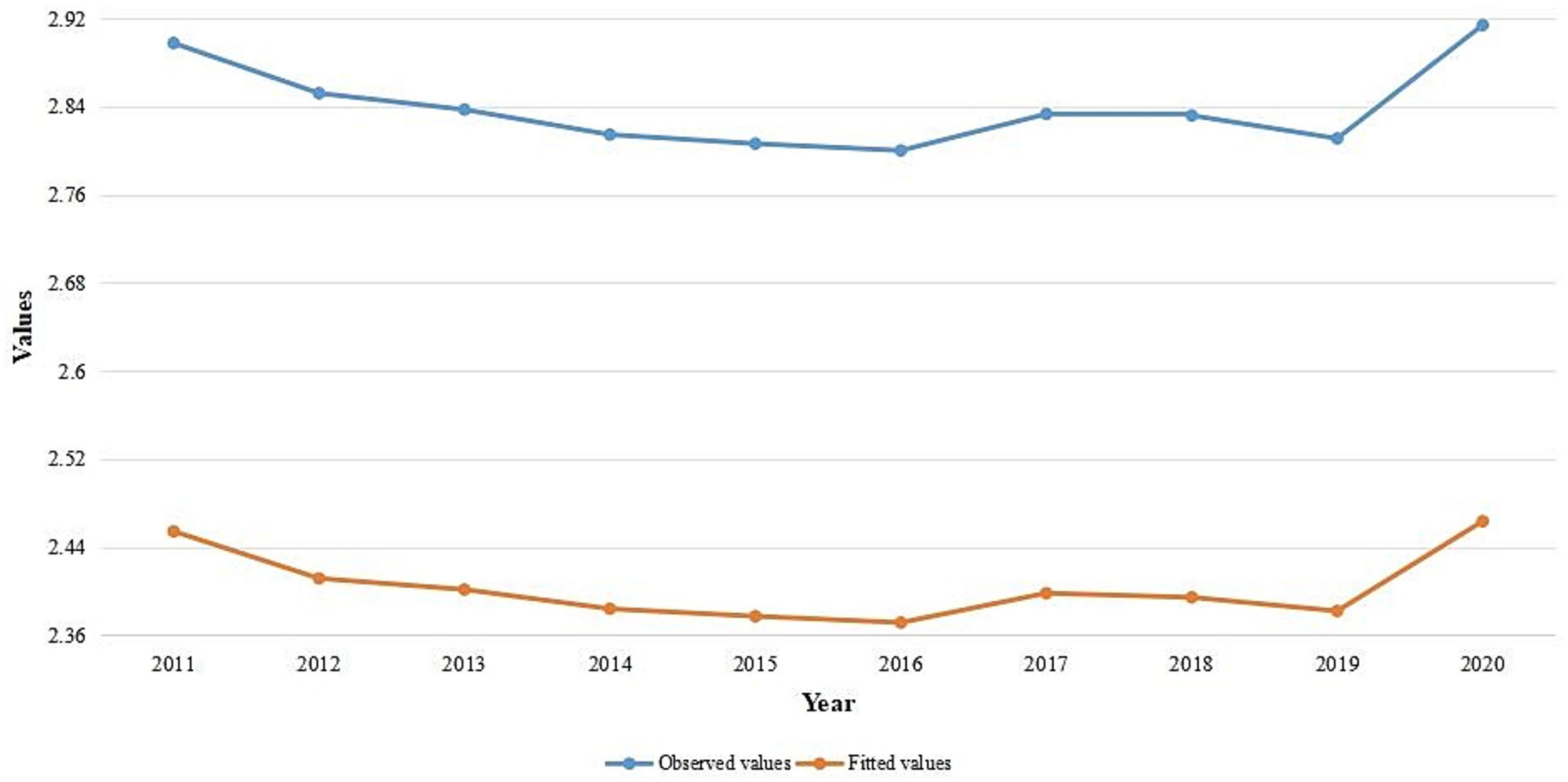
Temporal trend of the observed values and average fitted values for yearly malaria infection rates in SSA from 2011 to 2020.

Figure 9 shows the results of transformed observed values and raw overall trends in the SSA global, which may have been caused by trends in the intervention covariates. The trends of the observed values and fitted values look similar. According to the results, the proportion of malaria infections increased in 2017 after declining between 2011 and 2016. However, the pandemic caused the prevalence rates to rise in 2020 after declining between 2017 and 2019.

## 4 Discussion

According to this study, the average percentage of children infected with malaria fell from 21.3% in 2011 to 16.8% in 2016, with a small increase observed in 2017. However, it fell from an average of 16.9% in 2017 to 16.5% in 2019, before rising to an average of 17.8% in 2020 due to the coronavirus pandemic. Between 2011 and 2020, the proportion of malaria infections varied throughout all SSA states. According to Stonely (7), malaria transmission risk is high in West-central, Eastern, and West Africa, but low in the southern region of Africa due to the climatic network effect. In this study, the Northwest, Northeast, and certain regions of the eastern and southern states were low cluster areas, whereas the West-central, Central, and some Southeast states were SSA high cluster areas during the studied period. However, the South of Madagascar remained inconsequential, and the nations surrounding North Madagascar were low cluster areas.

According to Giardina et al. (19), while the overall risk of malaria has decreased in many SSA countries, there are high *parasitemia* clusters that enhance the estimated spatial variance, and the change in malaria risk varies substantially by location. The significance of fitting a spatial model is confirmed in this study by the estimate that the variation between locations is greater than the independent error variance. A moderate level of spatial correlation is indicated by the global spatial dependency parameter. The temporal correlation of the auto-regressive process in SSA is significant, and the temporal autocorrelation is greater than the spatial correlation.

According to Okumu (16), employing insecticide-treated mosquito nets can reduce exposure to malaria-carrying mosquitoes by 25–30%. According to this study, for every unit increase in the number of people sleeping under ITN annually, the SSA risk of malaria infection among children aged 2 to 10 will decrease by 34.07%. Avrakotos (32) states that the US President’s Malaria Initiative (PMI) works with countries to stop malaria by providing mosquito nets, spraying insecticides on dwellings, giving out preventative drugs, training medical professionals, and sponsoring malaria research. In addition to collaborating with community partners to promote regular mosquito net use, PMI has helped distribute 500 million insecticide-treated mosquito nets since 2005 (33). According to this study, the percentage of the population with access to ITN in their household during a given year will rise by a unit if the average annual rate of malaria infection in SSA increases by 34.54%. Additionally, if the SSA annual percentage of malaria cases increases by 55.08%, the percentage of people sleeping under ITN in homes with access to ITN will increase by a unit in a given year. Furthermore, if the SSA malaria infection rate increases by 1.32%, there will be one extra household covered by IRS in a given year. Besides, the percentage of malaria cases receiving effective antimalarial therapy will increase by one unit if the SSA yearly average malaria infection rate increases by 42.01%. The PMI website has featured a number of accomplishments made by its partner countries since its launch. Since 2006, the average number of malaria cases and deaths in PMI’s partner nations has decreased by 26 and 43%, respectively (14). According to this study, the states with the highest risk of malaria infection among children aged 2 to 10 in the SSA were West-central, Central, and a part Eastern. Nonetheless, the Northwest, Southern, and Horn of Africa states have the least chance of having a high malaria infection. According to this study, the states surrounding Equatorial Guinea, Cameroon, Angola, Congo, Gabon, Central African Republic, and Democratic Republic of Congo in the Central region, South Sudan, Uganda, Burundi, Zambia, Malawi, and Mozambique in the Eastern region, and Guinea, Sierra Leone, Liberia, Mali, Côte d’Ivoire, Burkina Faso, Ghana, Togo, Benin, Niger, and Nigeria in the Western region had the highest risk of malaria infection among children aged 2 to 10 years in the SSA during the study period. Although it showed an approximate decline in the average proportion of malaria infection in the SSA from 2011 to 2020, it started to rise in 2020 due to the COVID-19 Pandemic, which affected the whole health system globally (34, 35).

The limitations of this study should be considered when evaluating the findings. We omitted a number of risk factors that influence the prevalence of malaria infections in the SSA, including socioeconomic and meteorological conditions, because these variables were not in the dataset.

## 5 Conclusion

This study discovered that between 2011 and 2020, the effect of vector control activities on the rate of malaria cases varies by time and location in 634 states across 45 SSA nations. During the study period in the SSA, the estimated spatial autocorrelation is lower than the estimated temporal autocorrelation, while the estimated spatial variation is greater than the independent error variance. In this study, the malaria infections prevalence among children aged 2 to 10 decreased as the population’s use of ITN increased. For every increase in malaria cases among children between the ages of 2 and 10 in SSA, the percentage of people who have access to ITN in their homes would rise. Additionally, the proportion of families that have received IRS and the percentage of people sleeping under ITN -among those with access -would both increase. Moreover, the percentage of malaria cases that receive effective antimalarial medicine treatment would increase. Globally, the SSA annual malaria infection prevalence among children aged 2 to 10 decreased between 2011 and 2016, with a modest uptick observed in 2017. Nonetheless, during 2017 and 2019, the malaria infections prevalence decreased. Children aged 2 to 10 were most likely to get malaria in states located in the West-Central, Central, and Eastern regions. However, the Northwest, Southern, and Horn of Africa states have the lowest risk of having an elevated rate of infections with malaria. We recommend that the global end malaria councils and the malaria control and elimination program act in West-Central, Central, and some Eastern states to increase the number of interventions vector control and provide training on how to use it to reduce malaria risk in the region, because the greatest rates of infection with malaria in children between the ages of two and ten have been observed in these states.

## Data Availability

The datasets presented in this study can be found in online repositories. The names of the repository/repositories and accession number(s) can be found at: https://malariaatlas.org/.

## Footnotes

^1^ https://malariaatlas.org

^2^ www.gadm.org
