# Supplementary material for: Effects of vector control interventions on spatio-temporal changes of falciparum malaria risk in children aged 2–10 in sub-Saharan African regions during 2011–2020
Source: Front Public Health. 2025 Jul 2;13:1531771. doi: 10.3389/fpubh.2025.1531771 (PMC12263685; doi:10.3389/fpubh.2025.1531771)
Supplement: Supplementary file 1 [file Data_Sheet_1.docx]

**Supplemental files**


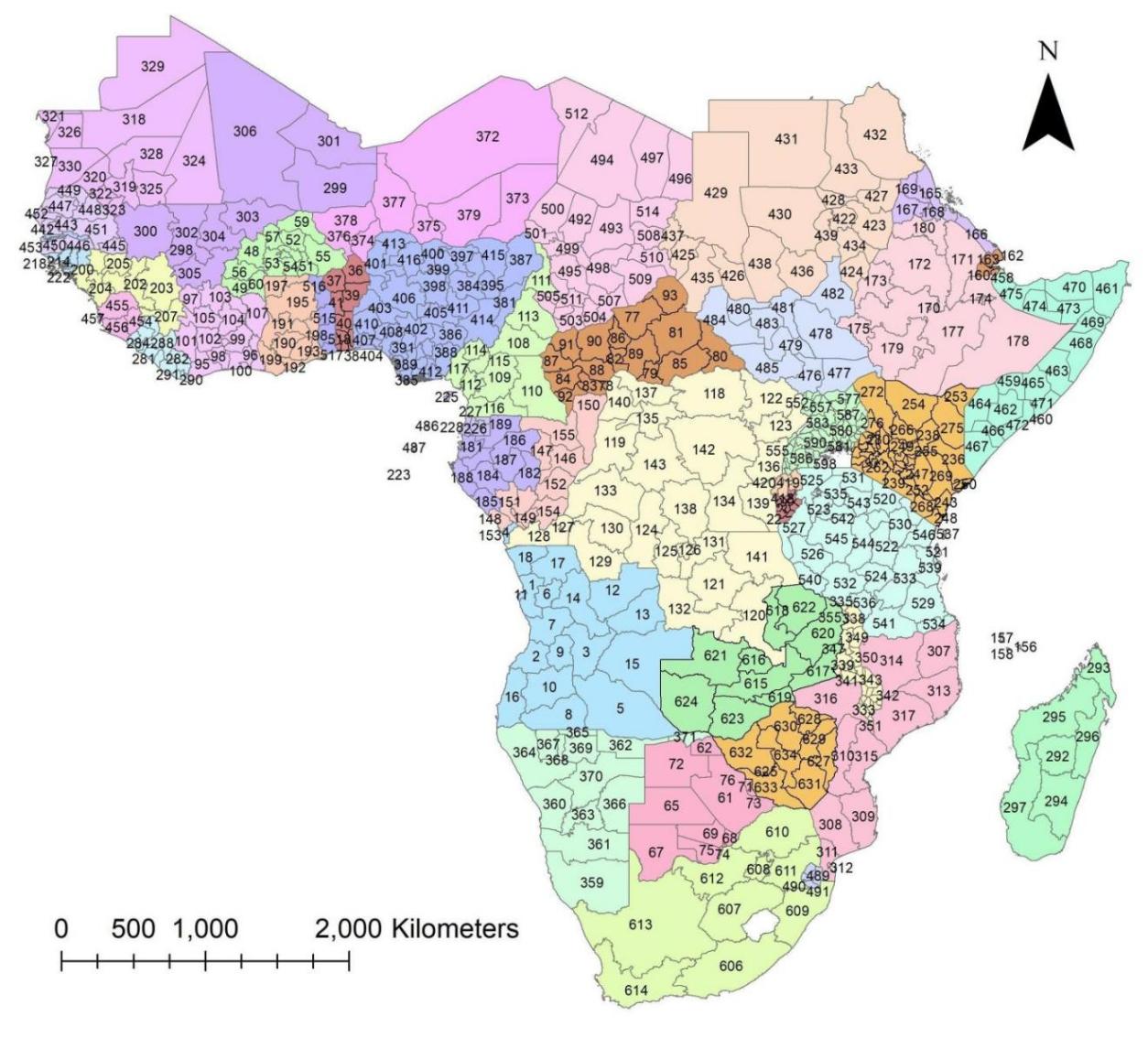


**Fig. S1**. Map of the study area at regional level and identification number. Source of shapefile: Database of Global Administrative Areas v.4.1 ([www.gadm.org](http://www.gadm.org)), own map output from ArcGIS v.10.8 (https://desktop.arcgis.com).

**Table S1**. The list of states identification number (SID) and states in the Sub-Saharan Africa corresponding with countries.

| **SID** | **State** | **Country** | **SID** | **State** | **Country** |
| --- | --- | --- | --- | --- | --- |
| 1 | Bengo | Angola | 264 | Nyandarua | Kenya |
| 2 | Benguela | Angola | 265 | Nyeri | Kenya |
| 3 | Bié | Angola | 266 | Samburu | Kenya |
| 4 | Cabinda | Angola | 267 | Siaya | Kenya |
| 5 | Cuando Cubango | Angola | 268 | Taita Taveta | Kenya |
| 6 | Cuanza Norte | Angola | 269 | Tana River | Kenya |
| 7 | Cuanza Sul | Angola | 270 | Tharaka-Nithi | Kenya |
| 8 | Cunene | Angola | 271 | Trans Nzoia | Kenya |
| 9 | Huambo | Angola | 272 | Turkana | Kenya |
| 10 | Huíla | Angola | 273 | Uasin Gishu | Kenya |
| 11 | Luanda | Angola | 274 | Vihiga | Kenya |
| 12 | Lunda Norte | Angola | 275 | Wajir | Kenya |
| 13 | Lunda Sul | Angola | 276 | West Pokot | Kenya |
| 14 | Malanje | Angola | 277 | Bomi | Liberia |
| 15 | Moxico | Angola | 278 | Bong | Liberia |
| 16 | Namibe | Angola | 279 | Gbapolu | Liberia |
| 17 | Uíge | Angola | 280 | Grand Cape Mount | Liberia |
| 18 | Zaire | Angola | 281 | GrandBassa | Liberia |
| 36 | Alibori | Benin | 282 | GrandGedeh | Liberia |
| 37 | Atakora | Benin | 283 | GrandKru | Liberia |
| 38 | Atlantique | Benin | 284 | Lofa | Liberia |
| 39 | Borgou | Benin | 285 | Margibi | Liberia |
| 40 | Collines | Benin | 286 | Maryland | Liberia |
| 41 | Donga | Benin | 287 | Montserrado | Liberia |
| 42 | Kouffo | Benin | 288 | Nimba | Liberia |
| 43 | Littoral | Benin | 289 | River Cess | Liberia |
| 44 | Mono | Benin | 290 | River Gee | Liberia |
| 45 | Ouémé | Benin | 291 | Sinoe | Liberia |
| 46 | Plateau | Benin | 292 | Antananarivo | Madagascar |
| 47 | Zou | Benin | 293 | Antsiranana | Madagascar |
| 61 | Central | Botswana | 294 | Fianarantsoa | Madagascar |
| 62 | Chobe | Botswana | 295 | Mahajanga | Madagascar |
| 63 | Francistown | Botswana | 296 | Toamasina | Madagascar |
| 64 | Gaborone | Botswana | 297 | Toliary | Madagascar |
| 65 | Ghanzi | Botswana | 331 | Balaka | Malawi |
| 66 | Jwaneng | Botswana | 332 | Blantyre | Malawi |
| 67 | Kgalagadi | Botswana | 333 | Chikwawa | Malawi |
| 68 | Kgatleng | Botswana | 334 | Chiradzulu | Malawi |
| 69 | Kweneng | Botswana | 335 | Chitipa | Malawi |
| 70 | Lobatse | Botswana | 336 | Dedza | Malawi |
| 71 | North-East | Botswana | 337 | Dowa | Malawi |
| 72 | North-West | Botswana | 338 | Karonga | Malawi |
| 73 | Selibe Phikwe | Botswana | 339 | Kasungu | Malawi |
| 74 | South-East | Botswana | 340 | Likoma | Malawi |
| 75 | Southern | Botswana | 341 | Lilongwe | Malawi |
| 76 | Sowa | Botswana | 342 | Machinga | Malawi |
| 48 | Boucle du Mouhoun | Burkina Faso | 343 | Mangochi | Malawi |
| 49 | Cascades | Burkina Faso | 344 | Mchinji | Malawi |
| 50 | Centre | Burkina Faso | 345 | Mulanje | Malawi |
| 51 | Centre-Est | Burkina Faso | 346 | Mwanza | Malawi |
| 52 | Centre-Nord | Burkina Faso | 347 | Mzimba | Malawi |
| 53 | Centre-Ouest | Burkina Faso | 348 | Neno | Malawi |
| 54 | Centre-Sud | Burkina Faso | 349 | Nkhata Bay | Malawi |
| 55 | Est | Burkina Faso | 350 | Nkhotakota | Malawi |
| 56 | Haut-Bassins | Burkina Faso | 351 | Nsanje | Malawi |
| 57 | Nord | Burkina Faso | 352 | Ntcheu | Malawi |
| 58 | Plateau-Central | Burkina Faso | 353 | Ntchisi | Malawi |
| 59 | Sahel | Burkina Faso | 354 | Phalombe | Malawi |
| 60 | Sud-Ouest | Burkina Faso | 355 | Rumphi | Malawi |
| 19 | Bubanza | Burundi | 356 | Salima | Malawi |
| 20 | Bujumbura Mairie | Burundi | 357 | Thyolo | Malawi |
| 21 | Bujumbura Rural | Burundi | 358 | Zomba | Malawi |
| 22 | Bururi | Burundi | 298 | Bamako | Mali |
| 23 | Cankuzo | Burundi | 299 | Gao | Mali |
| 24 | Cibitoke | Burundi | 300 | Kayes | Mali |
| 25 | Gitega | Burundi | 301 | Kidal | Mali |
| 26 | Karuzi | Burundi | 302 | Koulikoro | Mali |
| 27 | Kayanza | Burundi | 303 | Mopti | Mali |
| 28 | Kirundo | Burundi | 304 | Ségou | Mali |
| 29 | Makamba | Burundi | 305 | Sikasso | Mali |
| 30 | Muramvya | Burundi | 306 | Timbuktu | Mali |
| 31 | Muyinga | Burundi | 318 | Adrar | Mauritania |
| 32 | Mwaro | Burundi | 319 | Assaba | Mauritania |
| 33 | Ngozi | Burundi | 320 | Brakna | Mauritania |
| 34 | Rutana | Burundi | 321 | Dakhlet Nouadhibou | Mauritania |
| 35 | Ruyigi | Burundi | 322 | Gorgol | Mauritania |
| 108 | Adamaoua | Cameroon | 323 | Guidimaka | Mauritania |
| 109 | Centre | Cameroon | 324 | Hodh ech Chargui | Mauritania |
| 110 | Est | Cameroon | 325 | Hodh el Gharbi | Mauritania |
| 111 | Extrême-Nord | Cameroon | 326 | Inchiri | Mauritania |
| 112 | Littoral | Cameroon | 327 | Nouakchott | Mauritania |
| 113 | Nord | Cameroon | 328 | Tagant | Mauritania |
| 114 | Nord-Ouest | Cameroon | 329 | Tiris Zemmour | Mauritania |
| 115 | Ouest | Cameroon | 330 | Trarza | Mauritania |
| 116 | Sud | Cameroon | 307 | Cabo Delgado | Mozambique |
| 117 | Sud-Ouest | Cameroon | 308 | Gaza | Mozambique |
| 77 | Bamingui-Bangoran | Central African Republic | 309 | Inhambane | Mozambique |
| 78 | Bangui | Central African Republic | 310 | Manica | Mozambique |
| 79 | Basse-Kotto | Central African Republic | 311 | Maputo | Mozambique |
| 80 | Haute-Kotto | Central African Republic | 312 | Maputo City | Mozambique |
| 81 | Haut-Mbomou | Central African Republic | 313 | Nampula | Mozambique |
| 82 | Kémo | Central African Republic | 314 | Nassa | Mozambique |
| 83 | Lobaye | Central African Republic | 315 | Sofala | Mozambique |
| 84 | Mambéré-Kadéï | Central African Republic | 316 | Tete | Mozambique |
| 85 | Mbomou | Central African Republic | 317 | Zambezia | Mozambique |
| 86 | Nana-Grébizi | Central African Republic | 359 | !Karas | Namibia |
| 87 | Nana-Mambéré | Central African Republic | 360 | Erongo | Namibia |
| 88 | Ombella-M'Poko | Central African Republic | 361 | Hardap | Namibia |
| 89 | Ouaka | Central African Republic | 362 | Kavango | Namibia |
| 90 | Ouham | Central African Republic | 363 | Khomas | Namibia |
| 91 | Ouham-Pendé | Central African Republic | 364 | Kunene | Namibia |
| 92 | Sangha-Mbaéré | Central African Republic | 365 | Ohangwena | Namibia |
| 93 | Vakaga | Central African Republic | 366 | Omaheke | Namibia |
| 492 | Barh el Ghazel | Chad | 367 | Omusati | Namibia |
| 493 | Batha | Chad | 368 | Oshana | Namibia |
| 494 | Borkou | Chad | 369 | Oshikoto | Namibia |
| 495 | Chari-Baguirmi | Chad | 370 | Otjozondjupa | Namibia |
| 496 | Ennedi Est | Chad | 371 | Zambezi | Namibia |
| 497 | Ennedi Ouest | Chad | 372 | Agadez | Niger |
| 498 | Guéra | Chad | 373 | Diffa | Niger |
| 499 | Hadjer-Lamis | Chad | 374 | Dosso | Niger |
| 500 | Kanem | Chad | 375 | Maradi | Niger |
| 501 | Lac | Chad | 376 | Niamey | Niger |
| 502 | Logone Occidental | Chad | 377 | Tahoua | Niger |
| 503 | Logone Oriental | Chad | 378 | Tillabéry | Niger |
| 504 | Mandoul | Chad | 379 | Zinder | Niger |
| 505 | Mayo-Kebbi Est | Chad | 417 | Amajyaruguru | Rwanda |
| 506 | Mayo-Kebbi Ouest | Chad | 418 | Amajyepfo | Rwanda |
| 507 | Moyen-Chari | Chad | 419 | Iburasirazuba | Rwanda |
| 508 | Ouaddaï | Chad | 420 | Iburengerazuba | Rwanda |
| 509 | Salamat | Chad | 421 | Umujyi wa Kigali | Rwanda |
| 510 | Sila | Chad | 486 | Príncipe | Sao Tome and Principe |
| 511 | Tandjilé | Chad | 487 | São Tomé | Sao Tome and Principe |
| 512 | Tibesti | Chad | 440 | Dakar | Senegal |
| 513 | Ville de N'Djamena | Chad | 441 | Diourbel | Senegal |
| 514 | Wadi Fira | Chad | 442 | Fatick | Senegal |
| 156 | Mwali | Comoros | 443 | Kaffrine | Senegal |
| 157 | Njazídja | Comoros | 444 | Kaolack | Senegal |
| 158 | Nzwani | Comoros | 445 | Kédougou | Senegal |
| 380 | Abia | Nigeria | 446 | Kolda | Senegal |
| 381 | Adamawa | Nigeria | 447 | Louga | Senegal |
| 382 | Akwa Ibom | Nigeria | 448 | Matam | Senegal |
| 383 | Anambra | Nigeria | 449 | Saint-Louis | Senegal |
| 384 | Bauchi | Nigeria | 450 | Sédhiou | Senegal |
| 385 | Bayelsa | Nigeria | 451 | Tambacounda | Senegal |
| 386 | Benue | Nigeria | 452 | Thiès | Senegal |
| 387 | Borno | Nigeria | 453 | Ziguinchor | Senegal |
| 388 | Cross River | Nigeria | 454 | Eastern | Sierra Leone |
| 389 | Delta | Nigeria | 455 | Northern | Sierra Leone |
| 390 | Ebonyi | Nigeria | 456 | Southern | Sierra Leone |
| 391 | Edo | Nigeria | 457 | Western | Sierra Leone |
| 392 | Ekiti | Nigeria | 458 | Awdal | Somalia |
| 393 | Enugu | Nigeria | 459 | Bakool | Somalia |
| 394 | Federal Capital Territory | Nigeria | 460 | Banaadir | Somalia |
| 395 | Gombe | Nigeria | 461 | Bari | Somalia |
| 396 | Imo | Nigeria | 462 | Bay | Somalia |
| 397 | Jigawa | Nigeria | 463 | Galguduud | Somalia |
| 398 | Kaduna | Nigeria | 464 | Gedo | Somalia |
| 399 | Kano | Nigeria | 465 | Hiiraan | Somalia |
| 400 | Katsina | Nigeria | 466 | Jubbada Dhexe | Somalia |
| 401 | Kebbi | Nigeria | 467 | Jubbada Hoose | Somalia |
| 402 | Kogi | Nigeria | 468 | Mudug | Somalia |
| 403 | Kwara | Nigeria | 469 | Nugaal | Somalia |
| 404 | Lagos | Nigeria | 470 | Sanaag | Somalia |
| 405 | Nassarawa | Nigeria | 471 | Shabeellaha Dhexe | Somalia |
| 406 | Niger | Nigeria | 472 | Shabeellaha Hoose | Somalia |
| 407 | Ogun | Nigeria | 473 | Sool | Somalia |
| 408 | Ondo | Nigeria | 474 | Togdheer | Somalia |
| 409 | Osun | Nigeria | 475 | Woqooyi Galbeed | Somalia |
| 410 | Oyo | Nigeria | 422 | Al Jazirah | Sudan |
| 411 | Plateau | Nigeria | 423 | Al Qadarif | Sudan |
| 412 | Rivers | Nigeria | 424 | Blue Nile | Sudan |
| 413 | Sokoto | Nigeria | 425 | Central Darfur | Sudan |
| 414 | Taraba | Nigeria | 426 | East Darfur | Sudan |
| 415 | Yobe | Nigeria | 427 | Kassala | Sudan |
| 416 | Zamfara | Nigeria | 428 | Khartoum | Sudan |
| 144 | Bouenza | Congo | 429 | North Darfur | Sudan |
| 145 | Brazzaville | Congo | 430 | North Kurdufan | Sudan |
| 146 | Cuvette | Congo | 431 | Northern | Sudan |
| 147 | Cuvette-Ouest | Congo | 432 | Red Sea | Sudan |
| 148 | Kouilou | Congo | 433 | River Nile | Sudan |
| 149 | Lékoumou | Congo | 434 | Sennar | Sudan |
| 150 | Likouala | Congo | 435 | South Darfur | Sudan |
| 151 | Niari | Congo | 436 | South Kurdufan | Sudan |
| 152 | Plateaux | Congo | 437 | West Darfur | Sudan |
| 153 | Pointe Noire | Congo | 438 | West Kurdufan | Sudan |
| 154 | Pool | Congo | 439 | White Nile | Sudan |
| 155 | Sangha | Congo | 520 | Arusha | Tanzania |
| 118 | Bas-Uélé | DR Congo | 521 | Dar es Salaam | Tanzania |
| 119 | Équateur | DR Congo | 522 | Dodoma | Tanzania |
| 120 | Haut-Katanga | DR Congo | 523 | Geita | Tanzania |
| 121 | Haut-Lomami | DR Congo | 524 | Iringa | Tanzania |
| 122 | Haut-Uélé | DR Congo | 525 | Kagera | Tanzania |
| 123 | Ituri | DR Congo | 526 | Katavi | Tanzania |
| 124 | Kasaï | DR Congo | 527 | Kigoma | Tanzania |
| 125 | Kasaï-Central | DR Congo | 528 | Kilimanjaro | Tanzania |
| 126 | Kasaï-Oriental | DR Congo | 529 | Lindi | Tanzania |
| 127 | Kinshasa | DR Congo | 530 | Manyara | Tanzania |
| 128 | Kongo-Central | DR Congo | 531 | Mara | Tanzania |
| 129 | Kwango | DR Congo | 532 | Mbeya | Tanzania |
| 130 | Kwilu | DR Congo | 533 | Morogoro | Tanzania |
| 131 | Lomami | DR Congo | 534 | Mtwara | Tanzania |
| 132 | Lualaba | DR Congo | 535 | Mwanza | Tanzania |
| 133 | Maï-Ndombe | DR Congo | 536 | Njombe | Tanzania |
| 134 | Maniema | DR Congo | 537 | Pemba North | Tanzania |
| 135 | Mongala | DR Congo | 538 | Pemba South | Tanzania |
| 136 | Nord-Kivu | DR Congo | 539 | Pwani | Tanzania |
| 137 | Nord-Ubangi | DR Congo | 540 | Rukwa | Tanzania |
| 138 | Sankuru | DR Congo | 541 | Ruvuma | Tanzania |
| 139 | Sud-Kivu | DR Congo | 542 | Shinyanga | Tanzania |
| 140 | Sud-Ubangi | DR Congo | 543 | Simiyu | Tanzania |
| 141 | Tanganyika | DR Congo | 544 | Singida | Tanzania |
| 142 | Tshopo | DR Congo | 545 | Tabora | Tanzania |
| 143 | Tshuapa | DR Congo | 546 | Tanga | Tanzania |
| 94 | Abidjan | Côte d'Ivoire | 547 | Zanzibar North | Tanzania |
| 95 | Bas-Sassandra | Côte d'Ivoire | 548 | Zanzibar South and Central | Tanzania |
| 96 | Comoé | Côte d'Ivoire | 549 | Zanzibar West | Tanzania |
| 97 | Denguélé | Côte d'Ivoire | 606 | Eastern Cape | South Africa |
| 98 | Gôh-Djiboua | Côte d'Ivoire | 607 | Free State | South Africa |
| 99 | Lacs | Côte d'Ivoire | 608 | Gauteng | South Africa |
| 100 | Lagunes | Côte d'Ivoire | 609 | KwaZulu-Natal | South Africa |
| 101 | Montagnes | Côte d'Ivoire | 610 | Limpopo | South Africa |
| 102 | Sassandra-Marahoué | Côte d'Ivoire | 611 | Mpumalanga | South Africa |
| 103 | Savanes | Côte d'Ivoire | 612 | North West | South Africa |
| 104 | Vallée du Bandama | Côte d'Ivoire | 613 | Northern Cape | South Africa |
| 105 | Woroba | Côte d'Ivoire | 614 | Western Cape | South Africa |
| 106 | Yamoussoukro | Côte d'Ivoire | 476 | Central Equatoria | South Sudan |
| 107 | Zanzan | Côte d'Ivoire | 477 | Eastern Equatoria | South Sudan |
| 159 | Ali Sabieh | Djibouti | 478 | Jungoli | South Sudan |
| 160 | Dikhil | Djibouti | 479 | Lakes | South Sudan |
| 161 | Djibouti | Djibouti | 480 | North Bahr-al-Ghazal | South Sudan |
| 162 | Obock | Djibouti | 481 | Unity | South Sudan |
| 163 | Tadjourah | Djibouti | 482 | Upper Nile | South Sudan |
| 223 | Annobón | Equatorial Guinea | 483 | Warap | South Sudan |
| 224 | Bioko Norte | Equatorial Guinea | 484 | West Bahr-al-Ghazal | South Sudan |
| 225 | Bioko Sur | Equatorial Guinea | 485 | West Equatoria | South Sudan |
| 226 | Centro Sur | Equatorial Guinea | 488 | Hhohho | eSwatini |
| 227 | Kié-Ntem | Equatorial Guinea | 489 | Lubombo | eSwatini |
| 228 | Litoral | Equatorial Guinea | 490 | Manzini | eSwatini |
| 229 | Wele-Nzas | Equatorial Guinea | 491 | Shiselweni | eSwatini |
| 164 | Anseba | Eritrea | 208 | Banjul | Gambia |
| 165 | Debub | Eritrea | 209 | Lower River | Gambia |
| 166 | Debubawi Keyih Bahri | Eritrea | 210 | Maccarthy Island | Gambia |
| 167 | Gash Barka | Eritrea | 211 | North Bank | Gambia |
| 168 | Maekel | Eritrea | 212 | Upper River | Gambia |
| 169 | Semenawi Keyih Bahri | Eritrea | 213 | Western | Gambia |
| 170 | Addis Abeba | Ethiopia | 515 | Centre | Togo |
| 171 | Afar | Ethiopia | 516 | Kara | Togo |
| 172 | Amhara | Ethiopia | 517 | Maritime | Togo |
| 173 | Benshangul-Gumaz | Ethiopia | 518 | Plateaux | Togo |
| 174 | Dire Dawa | Ethiopia | 519 | Savanes | Togo |
| 175 | Gambela Peoples | Ethiopia | 550 | Adjumani | Uganda |
| 176 | Harari People | Ethiopia | 551 | Apac | Uganda |
| 177 | Oromia | Ethiopia | 552 | Arua | Uganda |
| 178 | Somali | Ethiopia | 553 | Bugiri | Uganda |
| 179 | SNNP | Ethiopia | 554 | Bundibugyo | Uganda |
| 180 | Tigray | Ethiopia | 555 | Bushenyi | Uganda |
| 181 | Estuaire | Gabon | 556 | Busia | Uganda |
| 182 | Haut-Ogooué | Gabon | 557 | Gulu | Uganda |
| 183 | Moyen-Ogooué | Gabon | 558 | Hoima | Uganda |
| 184 | Ngounié | Gabon | 559 | Iganga | Uganda |
| 185 | Nyanga | Gabon | 560 | Jinja | Uganda |
| 186 | Ogooué-Ivindo | Gabon | 561 | Kabale | Uganda |
| 187 | Ogooué-Lolo | Gabon | 562 | Kabarole | Uganda |
| 188 | Ogooué-Maritime | Gabon | 563 | Kaberamaido | Uganda |
| 189 | Wouleu-Ntem | Gabon | 564 | Kalangala | Uganda |
| 190 | Ashanti | Ghana | 565 | Kampala | Uganda |
| 191 | Brong Ahafo | Ghana | 566 | Kamuli | Uganda |
| 192 | Central | Ghana | 567 | Kamwenge | Uganda |
| 193 | Eastern | Ghana | 568 | Kanungu | Uganda |
| 194 | Greater Accra | Ghana | 569 | Kapchorwa | Uganda |
| 195 | Northern | Ghana | 570 | Kasese | Uganda |
| 196 | Upper East | Ghana | 571 | Katakwi | Uganda |
| 197 | Upper West | Ghana | 572 | Kayunga | Uganda |
| 198 | Volta | Ghana | 573 | Kibale | Uganda |
| 199 | Western | Ghana | 574 | Kiboga | Uganda |
| 200 | Boké | Guinea | 575 | Kisoro | Uganda |
| 201 | Conakry | Guinea | 576 | Kitgum | Uganda |
| 202 | Faranah | Guinea | 577 | Kotido | Uganda |
| 203 | Kankan | Guinea | 578 | Kumi | Uganda |
| 204 | Kindia | Guinea | 579 | Kyenjojo | Uganda |
| 205 | Labé | Guinea | 580 | Lira | Uganda |
| 206 | Mamou | Guinea | 581 | Luwero | Uganda |
| 207 | Nzérékoré | Guinea | 582 | Masaka | Uganda |
| 214 | Bafatá | Guinea-Bissau | 583 | Masindi | Uganda |
| 215 | Biombo | Guinea-Bissau | 584 | Mayuge | Uganda |
| 216 | Bissau | Guinea-Bissau | 585 | Mbale | Uganda |
| 217 | Bolama | Guinea-Bissau | 586 | Mbarara | Uganda |
| 218 | Cacheu | Guinea-Bissau | 587 | Moroto | Uganda |
| 219 | Gabú | Guinea-Bissau | 588 | Moyo | Uganda |
| 220 | Oio | Guinea-Bissau | 589 | Mpigi | Uganda |
| 221 | Quinara | Guinea-Bissau | 590 | Mubende | Uganda |
| 222 | Tombali | Guinea-Bissau | 591 | Mukono | Uganda |
| 230 | Baringo | Kenya | 592 | Nakapiripirit | Uganda |
| 231 | Bomet | Kenya | 593 | Nakasongola | Uganda |
| 232 | Bungoma | Kenya | 594 | Nebbi | Uganda |
| 233 | Busia | Kenya | 595 | Ntungamo | Uganda |
| 234 | Elgeyo-Marakwet | Kenya | 596 | Pader | Uganda |
| 235 | Embu | Kenya | 597 | Pallisa | Uganda |
| 236 | Garissa | Kenya | 598 | Rakai | Uganda |
| 237 | Homa Bay | Kenya | 599 | Rukungiri | Uganda |
| 238 | Isiolo | Kenya | 600 | Sembabule | Uganda |
| 239 | Kajiado | Kenya | 601 | Sironko | Uganda |
| 240 | Kakamega | Kenya | 602 | Soroti | Uganda |
| 241 | Kericho | Kenya | 603 | Tororo | Uganda |
| 242 | Kiambu | Kenya | 604 | Wakiso | Uganda |
| 243 | Kilifi | Kenya | 605 | Yumbe | Uganda |
| 244 | Kirinyaga | Kenya | 615 | Central | Zambia |
| 245 | Kisii | Kenya | 616 | Copperbelt | Zambia |
| 246 | Kisumu | Kenya | 617 | Eastern | Zambia |
| 247 | Kitui | Kenya | 618 | Luapula | Zambia |
| 248 | Kwale | Kenya | 619 | Lusaka | Zambia |
| 249 | Laikipia | Kenya | 620 | Muchinga | Zambia |
| 250 | Lamu | Kenya | 621 | North-Western | Zambia |
| 251 | Machakos | Kenya | 622 | Northern | Zambia |
| 252 | Makueni | Kenya | 623 | Southern | Zambia |
| 253 | Mandera | Kenya | 624 | Western | Zambia |
| 254 | Marsabit | Kenya | 625 | Bulawayo | Zimbabwe |
| 255 | Meru | Kenya | 626 | Harare | Zimbabwe |
| 256 | Migori | Kenya | 627 | Manicaland | Zimbabwe |
| 257 | Mombasa | Kenya | 628 | Mashonaland Central | Zimbabwe |
| 258 | Murang'a | Kenya | 629 | Mashonaland East | Zimbabwe |
| 259 | Nairobi | Kenya | 630 | Mashonaland West | Zimbabwe |
| 260 | Nakuru | Kenya | 631 | Masvingo | Zimbabwe |
| 261 | Nandi | Kenya | 632 | Matabeleland North | Zimbabwe |
| 262 | Narok | Kenya | 633 | Matabeleland South | Zimbabwe |
| 263 | Nyamira | Kenya | 634 | Midlands | Zimbabwe |

**SID**: it’s the states identification number


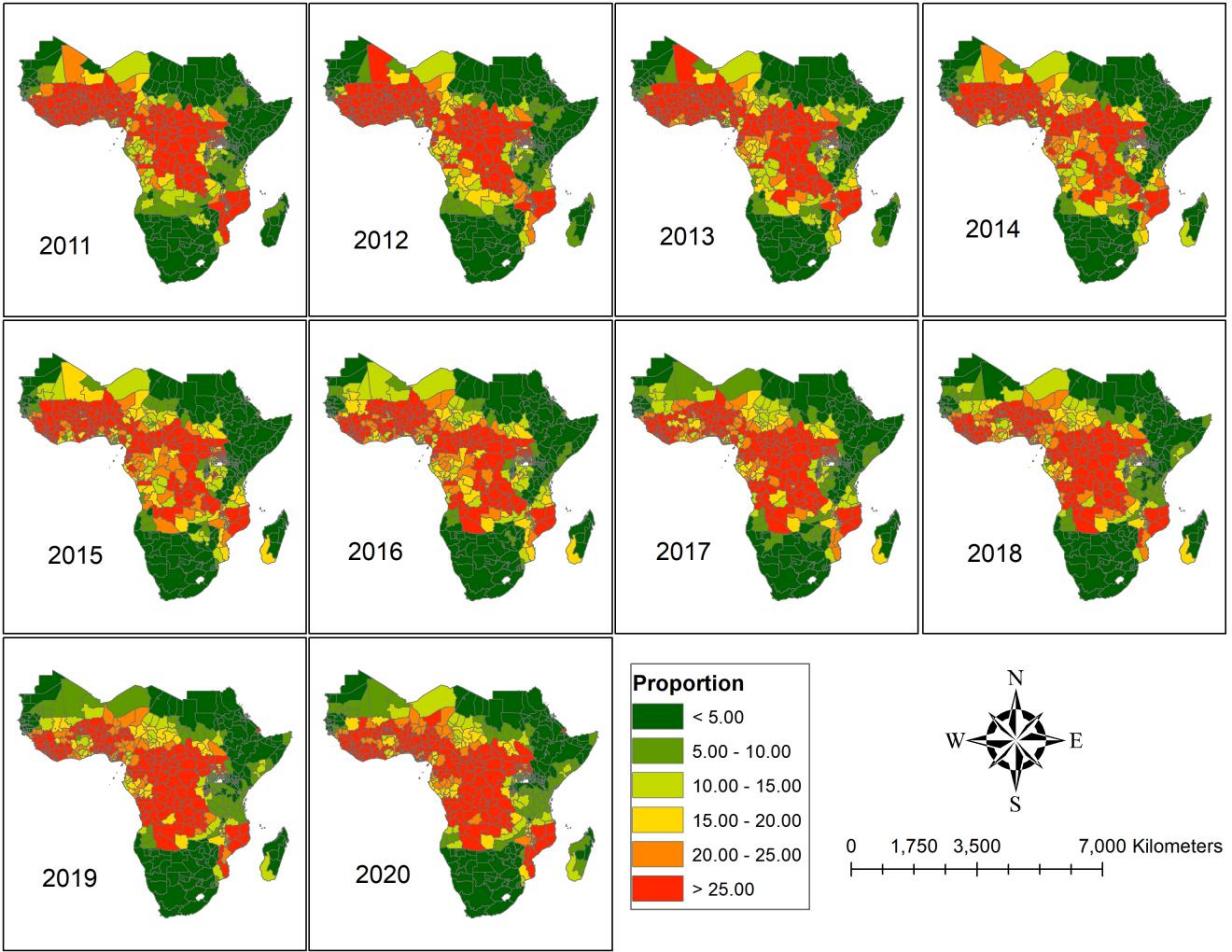


**Fig. S2** Spatiotemporal of the proportion of malaria infection for ${PfPR}_{2-10}$ in the SSA from 2011 to 2020. Source of shapefile: Database of Global Administrative Areas v.4.1 ([www.gadm.org](http://www.gadm.org)), own map output from ArcGIS v.10.8 (https://desktop.arcgis.com).


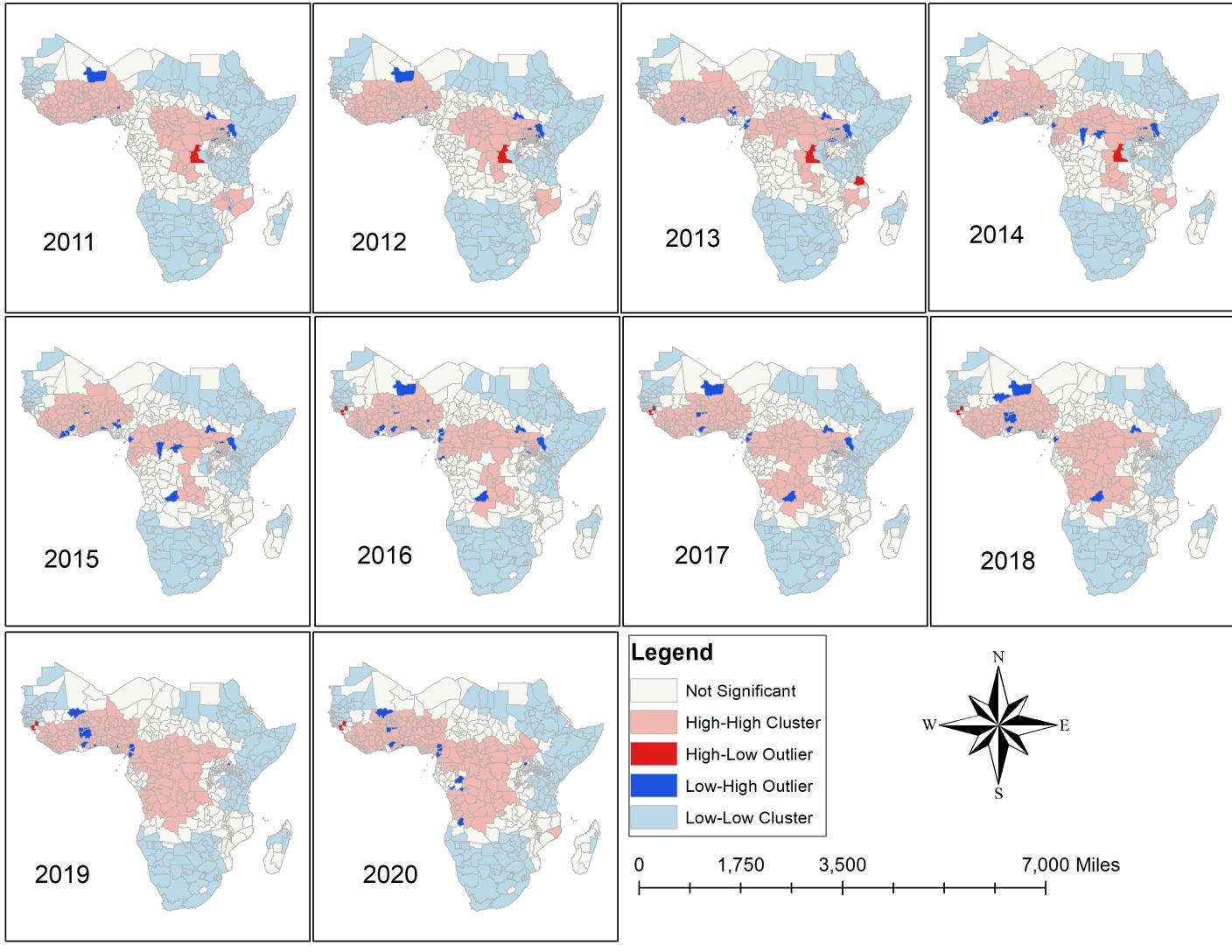


**Fig. S3**. Cluster and outlier analysis malaria infection for ${PfPR}_{2-10}$ in the SSA from 2011 to 2020. Source of shapefile: Database of Global Administrative Areas v.4.1 ([www.gadm.org](http://www.gadm.org)), own map output from ArcGIS v.10.8 (https://desktop.arcgis.com).


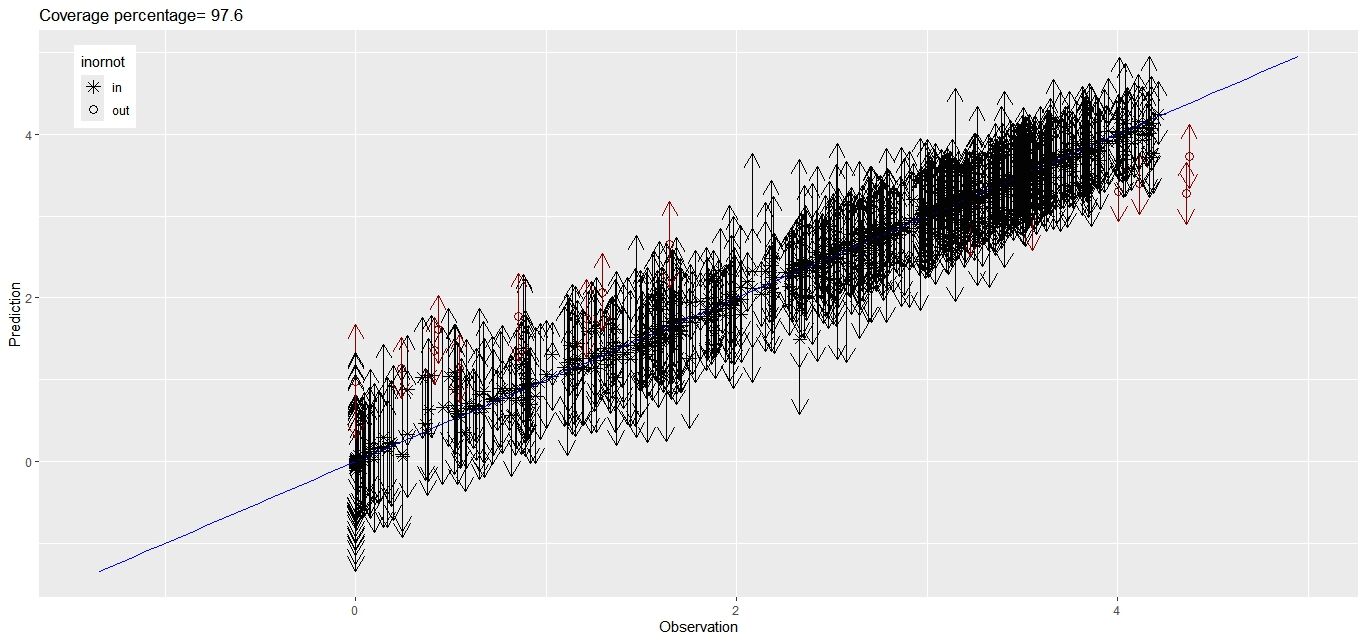


**Fig. S4**. Observed versus predicted plot for validations performed using the AR(1) model fitted


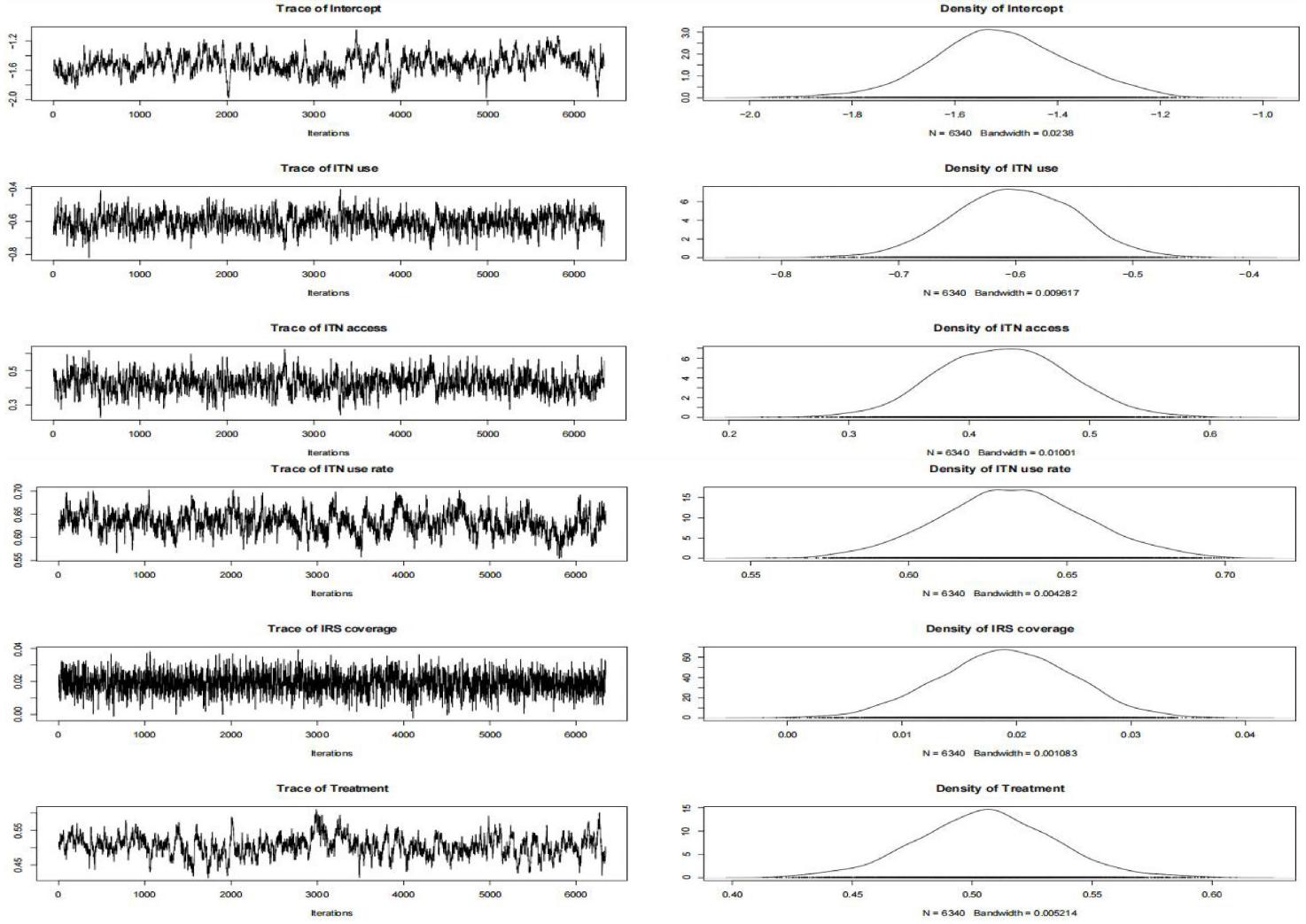


**Fig. S5**. Posterior distributions of the regression parameters ($\beta$)


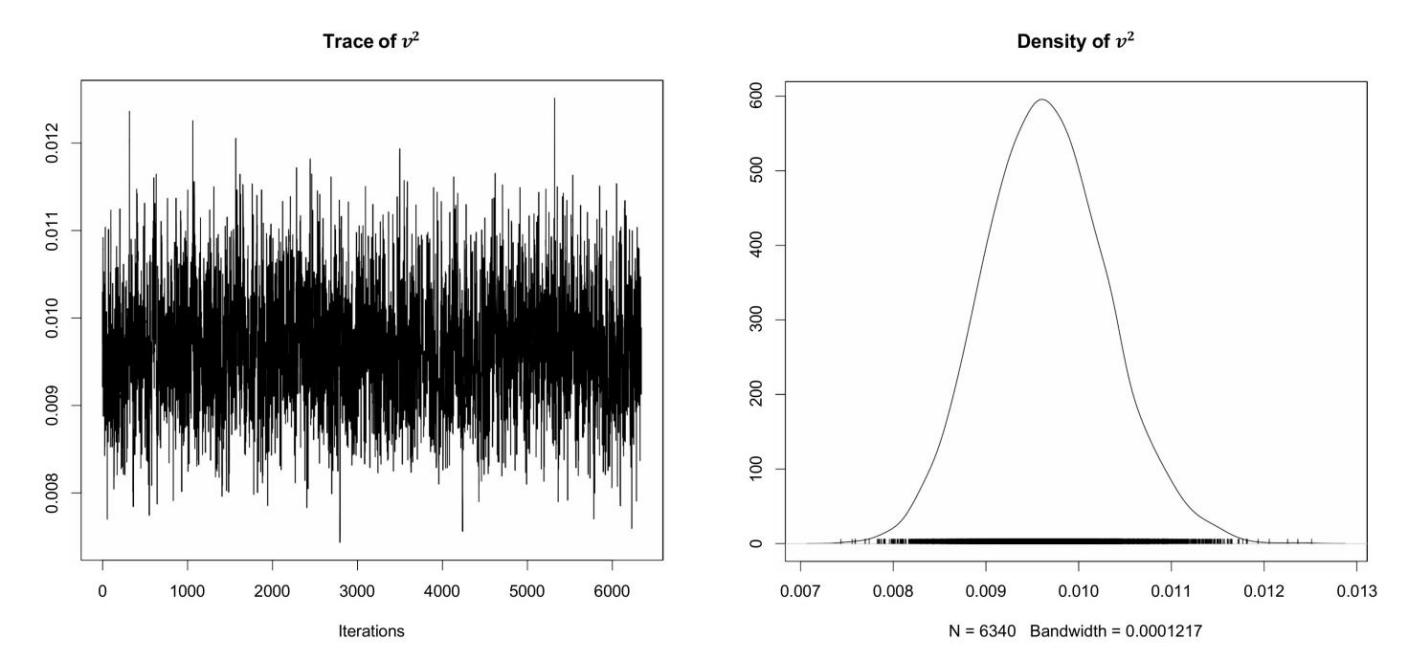


**Fig. S6**. Posterior distributions of the independent error variance


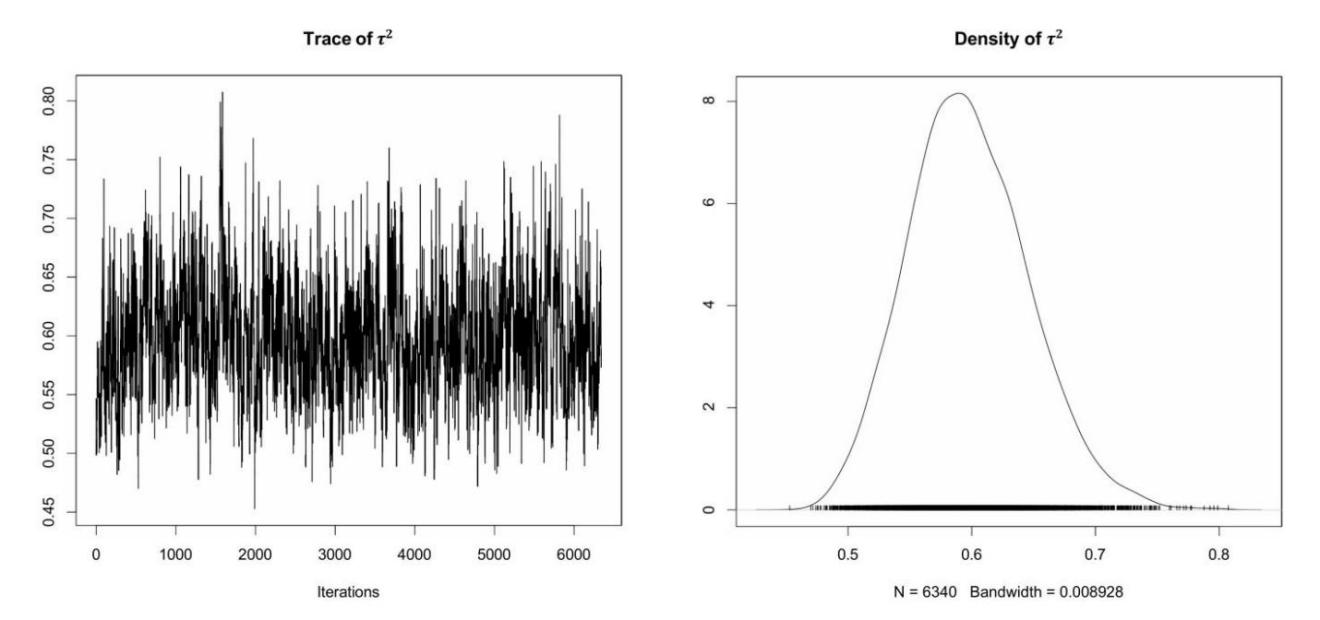


**Fig. S7**. Posterior distributions of the spatial variance


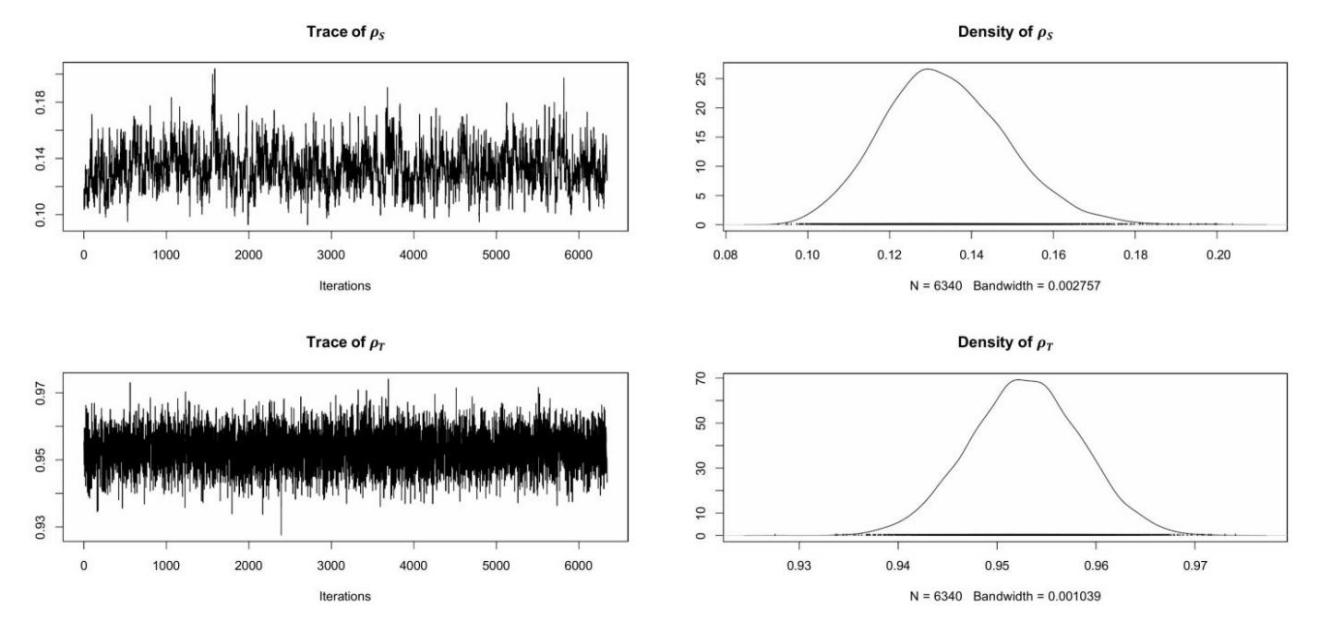


**Fig. S8**. Posterior distributions of the dependence parameters
